# Computational modeling suggests binding-induced expansion of Epsin disordered regions upon association with AP2

**DOI:** 10.1371/journal.pcbi.1008474

**Published:** 2021-01-06

**Authors:** N. Suhas Jagannathan, Christopher W. V. Hogue, Lisa Tucker-Kellogg

**Affiliations:** 1 Cancer & Stem Cell Biology, and Centre for Computational Biology, Duke-NUS Medical School, 8 College Road, Singapore; 2 Singapore-MIT Alliance, Computation and Systems Biology Program, National University of Singapore, Singapore; 3 Mechanobiology Institute, National University of Singapore, Singapore; Children's National Hospital, UNITED STATES

## Abstract

Intrinsically disordered regions (IDRs) are prevalent in the eukaryotic proteome. Common functional roles of IDRs include forming flexible linkers or undergoing allosteric folding-upon-binding. Recent studies have suggested an additional functional role for IDRs: generating steric pressure on the plasma membrane during endocytosis, via molecular crowding. However, in order to accomplish useful functions, such crowding needs to be regulated in space (e.g., endocytic hotspots) and time (e.g., during vesicle formation). In this work, we explore binding-induced regulation of IDR steric volume. We simulate the IDRs of two proteins from Clathrin-mediated endocytosis (CME) to see if their conformational spaces are regulated via binding-induced expansion. Using Monte-Carlo computational modeling of excluded volumes, we generate large conformational ensembles (3 million) for the IDRs of Epsin and Eps15 and dock the conformers to the alpha subunit of Adaptor Protein 2 (AP2α), their CME binding partner. Our results show that as more molecules of AP2α are bound, the Epsin-derived ensemble shows a significant increase in global dimensions, measured as the radius of Gyration (R_G_) and the end-to-end distance (EED). Unlike Epsin, Eps15-derived conformers that permit AP2α binding at one motif were found to be more likely to accommodate binding of AP2α at other motifs, suggesting a tendency toward co-accessibility of binding motifs. Co-accessibility was not observed for any pair of binding motifs in Epsin. Thus, we speculate that the disordered regions of Epsin and Eps15 perform different roles during CME, with accessibility in Eps15 allowing it to act as a recruiter of AP2α molecules, while binding-induced expansion of the Epsin disordered region could impose steric pressure and remodel the plasma membrane during vesicle formation.

## Author summary

Protein functions were originally believed to arise from ordered protein structures. This dogma was later challenged by the identification of intrinsically disordered proteins that lack specific structure. The functional roles of such proteins usually fell in two categories–exploiting the disorder for flexibility (like floppy connector), or imposing order upon binding to an external partner. In this study we explore the possibility of an alternative mechanism that harnesses disorder for function through *regulated* molecular crowding. Specifically, we use modeling to study two proteins involved in reshaping the cell membrane, Epsin and Eps15. We ask if they undergo binding-induced expansion, where binding of an external partner AP2 causes not a transition toward order, but rather an energetically favorable increase in propensity to occupy larger volumes. Our results show that Epsin tends to occupy a larger volume when bound to AP2, consistent with increased molecular crowding, which could help reshape the cell membrane. Such regulation of disorder via binding (without folding) opens hitherto unexplored avenues that cells might employ to harness disorder.

## Introduction

Cells typically internalize surface or external cargo through processes (e.g., endocytosis, phagocytosis, pinocytosis) that remodel the plasma membrane into cargo-containing vesicles. Clathrin-mediated endocytosis (CME) is one such cellular mechanism in which cell-surface cargo (typically membrane proteins and/or ligands) is internalized within a Clathrin-coated vesicle (CCV) that forms at the plasma membrane [1]. CME is a flexible process that can accommodate different sizes of cargo in vesicles ~60-120nm in size [2]. The overall CME process can be split into distinct temporal phases– 1) nucleation and initiation of the Clathrin-coated pit (CCP), 2) selection and binding of the cargo to the CCP, 3) growth and maturation of the CCV, 4) membrane scission and finally, 5) cytoplasmic uncoating. Different CME-related proteins are active during different phases of the process [2]. During phases 1, 2 and 3, participating proteins are believed to generate forces required to overcome membrane stiffness and surface tension, to form a membrane vesicle. Multiple studies have focused on the mechanistic details of such force generation, and common hypotheses include actin polymerization [3], scaffold-induced bending [4], and phase-separation of proteins [5]. However, no consensus yet exists. Recent studies have shown that an alternative source of force generation could be non-specific protein-protein crowding on the cytoplasmic face of the vesicle [6], especially by intrinsically disordered proteins (IDPs) [7]. Intriguingly, the CME proteome is highly enriched for proteins with intrinsically disordered regions (IDRs), and these IDRs contain binding motifs for other CME proteins [8]. Given that CME is tightly regulated and crucial for cell physiology, it is very likely that conserved CME-IDRs are functionally relevant, and exertion of steric pressure through molecular crowding is one way they could be functionally relevant.

IDRs are continuous stretches of amino acids (> 30 residues) that under native conditions do not fold into secondary structures [9–11]. Since their identification, IDRs and IDPs have been identified in cellular processes such as cell signaling, allosteric regulation, self-assembly, pathogenesis, post-translational modifications, alternative splicing, phase separation, and even extreme environment survival [12–19]. IDRs are now predicted to be part of more than 50% of the eukaryotic proteome [20] but are less prevalent in prokaryotes [21]. Typically, IDR sequences are depleted of hydrophobic residues and enriched with charged residues and proline. This profile of amino acids helps explain their lack of propensity for secondary structures. If the role of IDRs were primarily to provide linkers, their amino acid composition would be more important than their actual sequence. However, many IDRs are conserved across species at the sequence level, suggesting more specific functional roles. Underscoring this fact, IDRs in many systems have since been observed to undergo folding-upon-binding [22], and form secondary structures in response to binding by a partner. While folding-upon-binding is now agreed to be an important mode of IDP function, it may not be the only mode. An intriguing study by Busch *et*. *al*. [7] suggests that Epsin, a CME protein, contributes to membrane bending through steric pressure imposed as a result of molecular crowding of the disordered region. If membrane bending does indeed result from molecular crowding, then how would crowding be targeted and regulated?

We hypothesize that proteins involved in CME membrane bending have regulation of crowding and steric constraints induced by protein-protein binding. In this work, we perform *in silico* modelling of the disordered regions from two CME proteins, Epsin and Eps15, to see if steric hindrance and excluded volume might create circumstances in which successive binding to their partner protein AP2 may lead to an expansion of the conformational space occupied by the disordered regions (binding-induced expansion). If true, the regulation of expansion caused by AP2-binding may result in increased molecular crowding and higher steric pressure at endocytic hotspots, ultimately resulting in mechanical work and membrane bending.

Epsin is a CME protein that includes a long disordered C-terminal region (> 200 residues) known to contain multiple copies of a sequence motif that bind to the α subunit of the CME adaptor protein 2 (AP2α). Human Epsin isoforms are 576-640aa in length, and have an N-terminal domain called the Epsin N-terminal Homology domain (ENTH), that can insert into the membrane at an endocytic hotspot [23,24]. The ENTH domain is followed by an IDR ~400 residues in length that contains 8 copies of the sequence motif DPW (a known motif for binding AP2α, the alpha subunit of AP2). Finally, the C-terminus of Epsin contains binding sites for other CME proteins, such as Intersectin and Eps15 [8]. Previous studies have suggested that the Epsin IDR may induce membrane curvature through steric pressure [7]. While not implicated in these studies, we speculate that a second CME protein Eps15 might participate in similar mechanisms, owing to the many similarities it shares with Epsin: (1) Eps15 has a long (>200aa) C-terminal IDR, (2) Eps15 IDR has multiple binding sites to bind AP2α, and (3) the binding sites are very similar in sequence (DPW in Epsin vs DPF in Eps15). Human Eps15 (Epidermal growth factor receptor substrate 15) is an 896 aa protein that has been observed to accumulate near the rim of growing Clathrin coats [25]. Similar to Epsin, Eps15 has a structured N-terminal domain that binds other CME proteins such as Epsin, and this domain is followed by a long IDR (~350 residues) that contains 15 copies of the sequence motif DPF that binds AP2α [8]. Although there are multiple similarities between the disordered regions of Epsin and Eps15, there also exist differences (sequence length, composition, number of binding motifs, distribution of motifs in sequence etc.). Hence, in this study we apply excluded volume polymer models to the disordered regions of Epsin and Eps15 to compare their respective responses to AP2α binding, and to ask whether these disordered regions undergo AP2α binding-induced expansion.

Structural studies of IDRs are difficult due to unique challenges not present for folded structures–conformational heterogeneity, absence of secondary structure, flexible and dynamic structures, potential for aggregation. As a result, there are fewer experimental techniques available to study IDRs–the most common techniques used are NMR, Small Angle X-ray scattering (SAXS) and Single molecule fluorescence spectroscopy [26,27]. Hence computational tools provide an attractive alternative to study IDP behavior [28,29]. Computational methods typically represent IDPs as ensembles of structures (similar to NMR), and can vary by resolution (fine-grained vs coarse-grained) or modality/algorithms (e.g., Monte-Carlo vs MD simulations) [30–38]. While biophysical methods such as molecular dynamics (MD) provide a more accurate representation of biologically-feasible conformers (taking into account solvent behavior, energy minimization, etc.), they are computationally intensive and hence can only study smaller number of conformers. In contrast, Monte-Carlo (MC) methods can handle much larger ensembles, at the expense of resolution and accuracy for atomic-scale energetics. In this study, we use TraDES [33,39], a Monte-Carlo method that uses an excluded volume polymer model to generate large ensembles (3 million) of sterically-feasible conformers of the disordered regions from Epsin and Eps15 sequences. Hereafter, we call this model an Epsin-inspired Disordered Region (Epsin-iDR) and Eps15-inspired Disordered Region (Eps15-iDR). Using TraDES, we study how the characteristics of Epsin-iDR and Eps15-iDR vary, as a function of AP2α binding.

Our results show that compared to Eps15-iDR, steric constraints make it more difficult for Epsin-iDR to bind to AP2α (proportion of conformers capable of binding at a site). As a consequence, Eps15-iDR is capable of binding to more AP2α molecules simultaneously, and with each binding undergoes less reduction in available conformational space. Our results also show that the energetically-favorable subset of Epsin-iDR ensembles that allow increasing numbers of AP2α to bind, show a corresponding increase in dimensions (steric volume), suggesting binding-induced expansion of the Epsin-iDR. In contrast, the Eps15-iDR ensembles initially show a mild increase in dimensions upon AP2α binding, which is reversed when more molecules of AP2α are bound. In addition, the AP2α binding motifs of Eps15-iDR show a statistical tendency toward co-accessibility (AP2α binding at one motif increases the likelihood that other binding motifs in the same conformer are accessible to accommodate AP2α binding). This effect is not observed with the Epsin-iDR. Hence, we speculate that AP2α binding impacts the disordered regions of Epsin and Eps15 in different ways, which could lead to different functional roles. The binding-induced expansion of Epsin-iDRs can help impose steric pressure on the membrane (as suggested by other studies), whereas the observed tendency for co-occupancy of Eps15-iDRs may allow it to act as a recruiter of AP2α at the endocytic hotspot. Our work also suggests that statistical studies of IDP ensembles using simple excluded volume-based polymer structural models provide an effective means of generating hypotheses, and comparing/prioritizing IDPs for further experimental studies.

## Results

### The C-terminal regions of Epsin and Eps15 are disordered and evolutionarily conserved

**Fig 1:**
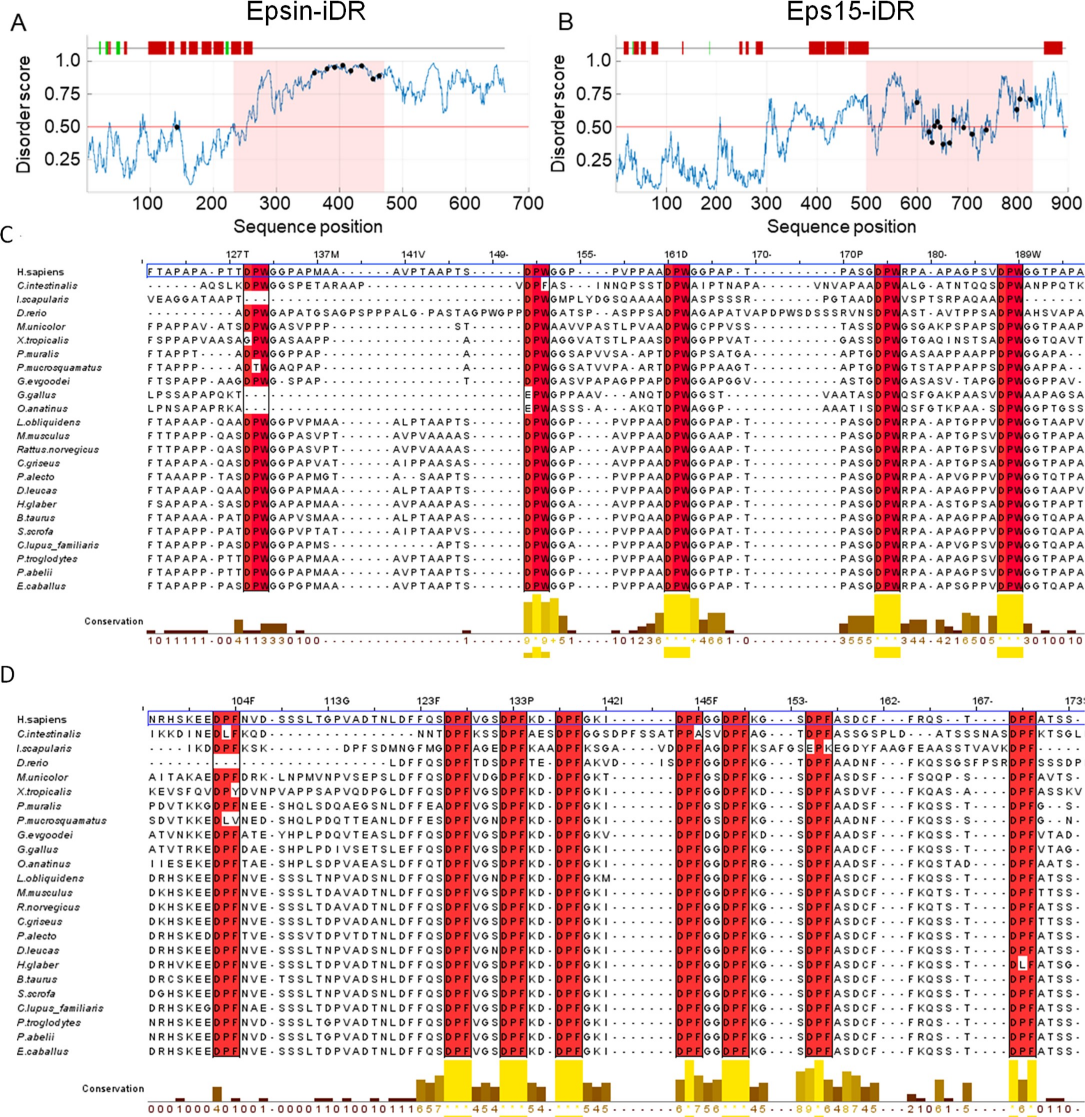
The C-terminal ends of Epsin and Eps15 are disordered and conserved. (A-B) The disorder propensity for each residue in either the Epsin (A, Uniprot ID: Q9Y6I3-1) sequence or the Eps15 (B, Uniprot ID: P42566) sequence was predicted using IUPred. Residues with predicted propensities greater than 0.5 (horizontal red line) are considered disordered. Secondary structure predictions of the respective sequences using JPred4 can be seen above the disorder plots. Along the top of (A-B), red vertical bars indicate predicted helix, green vertical bars indicate predicted sheets, and absence of vertical bars indicates a prediction of having no secondary structure. The disorder score is plotted in blue, and black markers on the plot indicate the beginning of the AP2α-binding DPW/DPF motifs. The pink shaded region represents the chosen sub-regions (Epsin-iDR and Eps15-iDR) that were used for subsequent analyses. (C-D) Multiple sequence alignments showing conservation of AP2α-binding motifs (DPW for Epsin-iDR in C, and DPF for Eps15-iDR in D) across multiple eukaryotic species, with human boxed in blue. Vertical rectangles indicate the location of the motifs in the human sequence and individual residues in the vertical rectangles are shaded red if they share the same residue as human. The conservation score below each alignment is a score in the range 0 (lowest) to 11 (highest, indicated as *), that reflects the conservation of physico-chemical properties of each amino acid column [42]. A short region of the full MSA is shown here. The full alignment and a list of the chosen species can be found in S1 Text.

We applied the disorder prediction tool IUPred and the secondary structure prediction tool JPred to sequences of human Epsin (Uniprot: Q9Y6I3-1) and Eps15 (Uniprot: P42566). In both cases, the tools suggested the presence of long C-terminal regions predicted to be disordered (Fig 1A and 1B). This is consistent with previous reports that have used circular dichroism and electron microscopy to detect intrinsic disorder in the C-termini of Epsin and Eps15 [40,41]. IUPred predicts that Epsin has a continuous disordered region from residue 253–662 (C terminus), whereas Eps15 shows a disordered region from 350–896 (C-terminus) interrupted by a few islands of residues with low propensities for disorder, but without any secondary structure in JPred predictions (Fig 1A and 1B). Interestingly, both Epsin and Eps15 disordered regions are starkly conserved in many species, from insects to human, especially at the AP2α binding sites (Fig 1C and 1D and S1 Text). Since we are primarily interested in exploring possible functions of these conserved disordered regions and the impact of sterics on conformational accessibility, we worked only with the following defined regions from the Epsin and Eps15 sequences. We chose these regions ensuring that the they included a predicted N-terminal helical region (used later to align conformers), are predicted to be mostly disordered elsewhere, and include all AP2α binding sites. The chosen subsequences correspond to the regions 232–471 from Epsin, and 498–830 from Eps15 (Fig 1A and 1B), and we define these as the Epsin-inspired disordered region (Epsin-iDR) and the Eps15-inspired disordered region (Eps15-iDR), respectively.

Analysis of the iDR regions using CIDER [43] shows that sequence parameters such as the fraction of charged residues (FCR), net charge per residue (NCPR) and charge patterning (Kappa parameter [44]) are comparable for both iDR sequences (Table 1). In the disorder phase space (fraction of positively vs. negatively charged residues), both sequences lie in the Globule-tadpole region (S2 Text), but very close to the phase region for Janus sequences (context-dependent collapsed or extended). This suggests that each of Epsin-iDR and Eps15-iDR ensembles may have biophysical characteristics that allow them to occupy collapsed or extended conformational spaces, depending on context.

**Table 1:** Sequence characteristics of the Epsin-iDR and Eps15-iDR sequences.

Features	Epsin-iDR	Eps15-iDR
Length (residues)	240	333
Fraction negatively charged (f_-_)	0.150	0.162
Fraction positively charged (f_+_)	0.083	0.066
Fraction charged residues (FCR)	0.233	0.228
Net charge per residue (NCPR)	-0.067	-0.096
Kappa (κ) parameter	0.123	0.132
Omega (Ω) parameter	0.119	0.190
Hydropathy	3.831	3.845

### Generation of conformational ensembles for Epsin-iDR and Eps15-iDR

To generate ensembles of sterically-feasible conformers for the Epsin-iDR and Eps15-iDR sequences, we used the FoldTraj program of the TraDES package [33,39], a tool that works via Monte-Carlo conformational sampling. TraDES builds conformers by performing a random walk through the allowed dihedral-angle space for each Cα in the sequence, and picking rotamers at random for each amino acid. Where required, users can impose constraints on particular amino acids by providing exact φ and ψ Ramachandran angles, or constraining them to adopt a helix, sheet or a coil structure. TraDES performs backtracking for error-correction, and generates conformers that do not have inter-atomic steric clashes and are hence considered sterically-feasible.

**Fig 2:**
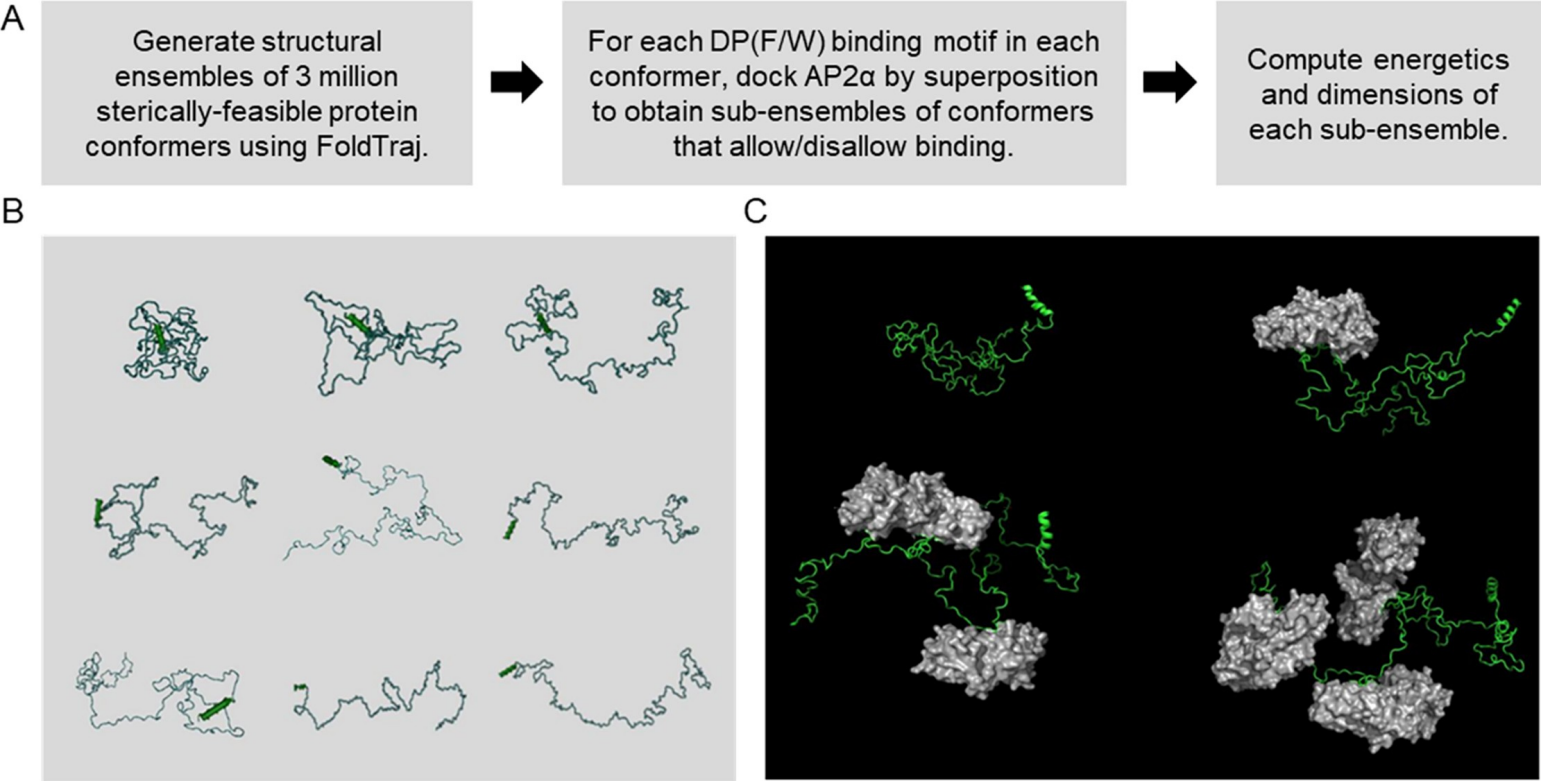
Generating the ensembles of Epsin-iDR and Eps15-iDR using TraDES. (A) The workflow used to generate and study the conformational ensembles of Epsin-iDR and Eps15-iDR. (B) Examples of some Epsin-iDR conformers generated using TraDES, showing a spectrum of sequences from very compact to very extended. (C) Examples of an unbound Epsin-iDR conformer and conformers that allow docking-by-superposition of 1, 2, and 3 copies of AP2α.

In our case, we first constrained the N-terminal residues of Epsin-iDR and Eps15-iDR (predicted helical regions) to adopt only helical dihedral angles. Next, we constrained every DPW motif in the Epsin-iDR to have the same φ and ψ angles experimentally observed for those residues in the PDB structure 1KY6 (AP2α bound to DPW peptide from Epsin). Similarly, all DPF motifs in Eps15 were constrained to have the same dihedral angles observed for those residues in the PDB structure 1KYF. We then used TraDES to generate 3 million conformers for both Epsin-iDR and Eps15-iDR, that we henceforth, we refer to as the full ensembles (Fig 2).

### Ensembles of Epsin-iDR conformers that bind more molecules of AP2α have larger dimensions

**Fig 3:**
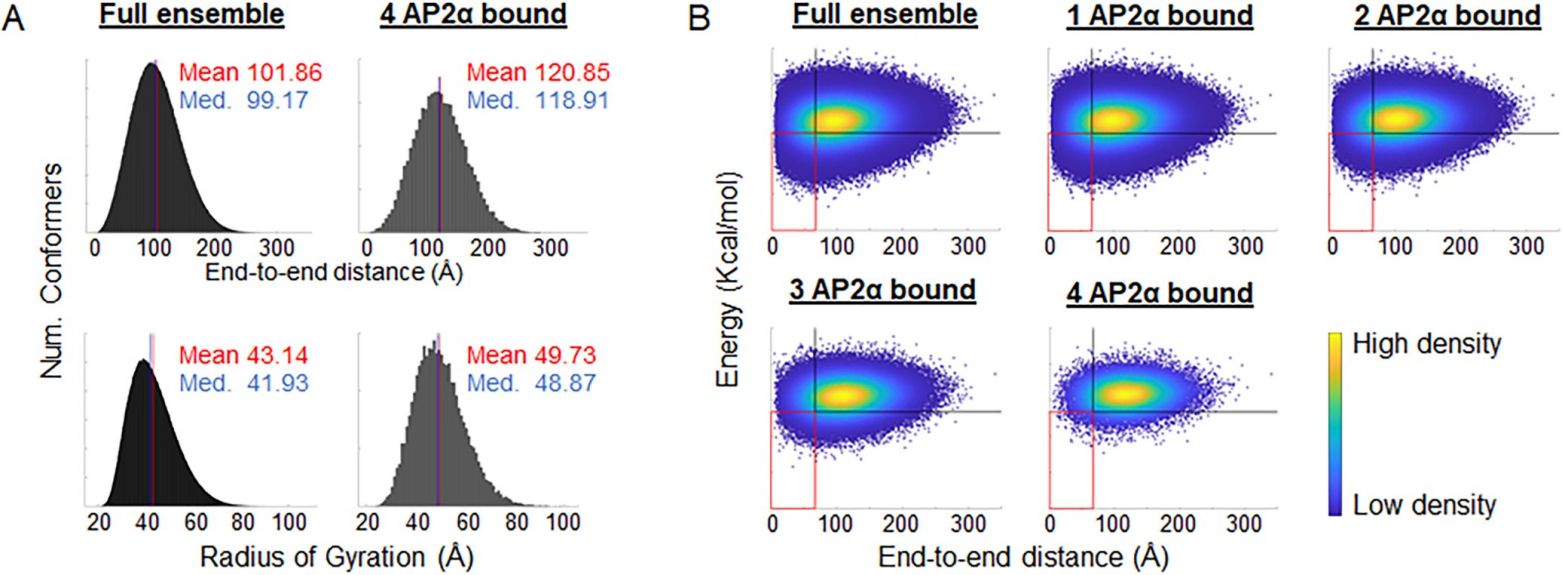
Epsin-iDR shows an energetically-favorable increase in dimensions upon AP2α binding. (A) Comparison of the end-to-end distances (EED, *top*) and Radii of gyration (R_G,_ *bottom*) between the full Epsin-iDR ensemble (*left*) and the 4 AP2α-bound sub-ensemble (*right*). The 4-bound sub-ensemble is right-shifted compared to the full ensemble for both EED and R_G_. (B) Density scatter plots of individual Epsin-iDR conformers in the EED vs. energy landscape. Energies were computed as per [45]. Within each plot, the vertical and horizontal black lines represent the 20^th^ percentile thresholds for low/high EED and low/high energy, respectively. The red rectangle at the lower left of each plot represents the group of compact low-energy conformers. As more molecules of AP2α are bound, there is a preferential depletion of conformers within the red rectangle.

We computed the dimensions of each conformer in the full Epsin-iDR ensemble using two metrics–EED (end-to-end distance, the distance from the N to the C-terminus), and R_G_ (radius of gyration, the root-mean-squared distance of all atoms to the centroid). As expected, the R_G_ distribution of the full ensemble (43.14 ± 9.71 Å) was larger than the R_G_ that would be expected for a folded protein of comparable length (~20 Å for 200 aa), confirming that the disordered nature results in mostly extended conformers. However, histograms (Fig 3A) show that the ensemble also includes compact structures. To see which members of the full ensemble accommodate AP2α binding, we docked AP2α (one at a time) to each DPW binding motif in each member of the full ensemble and considered the docking to be successful if the number of inter-chain atom clashes was less than 100 (see methods). From the list of conformers that could bind to at least one AP2α molecule (1-bound ensemble), and the list of conformers that could bind at least two AP2α molecules simultaneously (2-bound ensemble), we inferred individual sub-ensembles of Epsin-iDR that could bind to higher orders of AP2α molecules. Table 2 suggests that sub-ensembles of Epsin-iDR that allowed more molecules of AP2α to bind, showed increased EED and R_G_. With 4 molecules of AP2α bound simultaneously, Epsin-iDR ensembles showed an overall increase of ~19 Å in EED and ~6.6 Å in R_G_, suggesting global expansion of Epsin-iDR upon AP2α binding.

**Table 2:** Statistics of radius of gyration (R_G_) and end-to-end distance (EED) for the Epsin-iDR ensembles that allow binding to increasing numbers of AP2α molecules. Atom clash threshold = 100.

Ensemble	Total conformers	Average conformers	Std. dev conformers	Radius of Gyration (R_G_)		End-to-end distance (EED)	
				Mean	Std. Dev	Mean	Std. Dev
Full ensemble	3000000	3000000	0	43.14	9.71	101.86	39.81
1-bound	2328063	518359.88	128045.35	43.83	9.75	103.93	40.02
2-bound	1082362	73762.64	27114.07	45.30	9.86	108.19	40.58
3-bound	270080	8443.57	3938.72	47.31	9.98	113.93	41.28
4-bound	34872	764.34	417.37	49.73	10.10	120.85	42.13

S3 Text includes tables that show the sub-ensemble size (number of conformers), and dimensions (R_G_, EED) obtained when using either 50 or 150 for the atom clash threshold to define successful docking.

### Ensembles of Epsin-iDR that allow binding of more molecules of AP2α undergo selective depletion of compact low-energy conformers

We next considered the relative energies of the individual conformers to understand which conformers in the ensemble would be more likely to exist. To measure conformer energies, we used the method in [45]. We then plotted the location of each conformer in the landscape of EED vs. energy (hereafter called the EED-energy space) and classified the conformer as belonging to one of 4 regions in this EED-energy space (low/high EED, low/high energy). For both the EED and energy axes, we fixed the threshold between low and high at the 20^th^ percentile value along that axis, in the full ensemble (Fig 3B). As more molecules of AP2α were bound, the resulting sub-ensembles get progressively depleted of structures. Surprisingly, we observed that this depletion was not uniform across all four regions in the EED-energy space (Table 3). As more molecules of AP2α were bound, the fraction of high-EED high-energy structures increased. Comparing the full ensemble and the 4-bound ensemble, we observed that the compact low-energy structures go down in proportion from 5% to 1.1% (a change of -78%) whereas for the other three quadrants, the changes are -42%, +28% and -52%. If one accepts the approximation of sterically-feasible structures for mimicking energetically-feasible structures, then our results suggest that as more molecules of AP2α were bound, the region with compact, low-energy structures was getting depleted of structures faster than the other regions, suggesting that with increased AP2α binding, there were fewer and fewer stable compact conformations available.

**Table 3:** Relative proportions (in fractions) of Epsin-iDR conformers in different regions of the Energy–EED (end-to-end distance) state space. Thresholds for EED and Energy were set at the 20^th^ percentile of the corresponding values in the full ensemble.

Ensemble	Low EED Low Energy	Low EED High Energy	High EED High Energy	High EED Low Energy
Full ensemble	0.05	0.15	0.65	0.15
1-bound	0.043	0.142	0.674	0.141
2-bound	0.031	0.127	0.72	0.122
3-bound	0.02	0.108	0.775	0.097
4-bound	0.011	0.086	0.832	0.071

S4 Text includes tables that show the relative proportions of Epsin-iDR conformers in different quadrants, when using either 50 or 150 as the atom clash threshold to define successful docking.

### Ensembles of Eps15-iDR conformers that bind more molecules of AP2α show less binding-induced expansion than Epsin-iDR

Similar to Epsin-iDR, we docked AP2α by superposition to each of the 15 DPF motifs of the Eps15-iDR and defined a successful docking as having inter-chain atom clashes less than 100. While the Epsin-iDR ensemble (3 million) was depleted of structures with 4 molecules of AP2α bound, the Eps15-iDR ensemble was able to bind up to 10 AP2α molecules simultaneously. In contrast to Epsin-iDR, the Eps15-iDR shows a smaller increase in dimensions upon AP2α binding. Comparing the full Eps15-iDR ensemble against the 10-bound ensembles shows no increase in either R_G_ or EED (Table 4). Results corresponding to using 50 or 150 for atom clash threshold can be found in S5 Text.

**Table 4:** Statistics of radius of gyration (R_G_) and end-to-end distance (EED) for the Eps15-iDR ensembles that allow binding to increasing numbers of AP2α molecules. Atom clash threshold = 100.

Ensemble	Total conformers	Average conformers	Std. dev conformers	Radius of Gyration (R_G_)		End-to-end distance (EED)	
				Mean	Std. Dev	Mean	Std. Dev
Full Ensemble	3000000	3000000	0	53.80	12.20	124.84	50.80
1-bound	2985795.00	1017107.20	273962.56	53.82	12.20	124.93	50.79
2-bound	2872623.00	356937.40	113390.28	54.03	12.20	125.59	50.83
3-bound	2513001.00	141367.15	41644.90	54.65	12.23	127.49	51.07
4-bound	1877100.00	67087.08	15808.04	55.76	12.36	130.78	51.64
5-bound	1157941.00	37765.85	7035.57	57.11	12.61	134.70	52.50
6-bound	608470.00	23929.77	3737.67	57.98	13.00	137.12	53.47
7-bound	306707.00	16344.74	2213.93	57.41	13.32	135.39	53.98
8-bound	173845.00	11787.10	1382.39	55.68	13.07	130.42	52.92
9-bound	111030.00	8908.04	884.39	54.44	12.56	126.92	51.58
10-bound	72006.00	7033.33	572.34	54.05	12.30	125.76	50.98

We also computed the energies of all Eps15-iDR conformers using [45]. Conformers were classified into four regions of the EED-energy space as before (with thresholds set at the 20^th^ percentile value of the Eps15-iDR full ensemble). Density plots similar to Fig 3B for Eps15-iDR can be found in S6 Text. As with the R_G_ and EED measurements, we observed that the results are similar qualitatively (but weaker quantitatively) to Epsin-iDR until 5 molecules of AP2α were bound to Eps15-iDR. Further binding of AP2α reverses this trend. (Table 5, S7 Text). Hence the response of Eps15-iDR to AP2α binding appears to vary from mild to none depending on the number of AP2α bound. Hence, in order to obtain further insight into which states (1-bound, 2-bound etc.) are likely, it is also necessary to study whether binding of an AP2α molecule makes it easier or more difficult for Epsin/Eps15-iDR conformers to bind additional AP2α molecules.

**Table 5:** Relative proportions (in fractions) of Eps15-iDR conformers in different regions of the Energy–EED (end-to-end distance) state space. Thresholds for EED and Energy were set at the 20^th^ percentile of the corresponding values in the full ensemble.

Ensemble	Low EED Low Energy	Low EED High Energy	High EED High Energy	High EED Low Energy
Full ensemble	0.05	0.15	0.65	0.15
1-bound	0.049	0.15	0.65	0.15
2-bound	0.048	0.148	0.655	0.149
3-bound	0.044	0.142	0.669	0.146
4-bound	0.038	0.132	0.69	0.14
5-bound	0.033	0.122	0.713	0.133
6-bound	0.031	0.116	0.725	0.128
7-bound	0.035	0.123	0.71	0.131
8-bound	0.042	0.137	0.681	0.14
9-bound	0.046	0.145	0.662	0.146
10-bound	0.048	0.148	0.657	0.147

### AP2α binding motifs in Eps15-iDR (but not Epsin-iDR) show a statistical tendency toward co-accessibility

**Fig 4:**
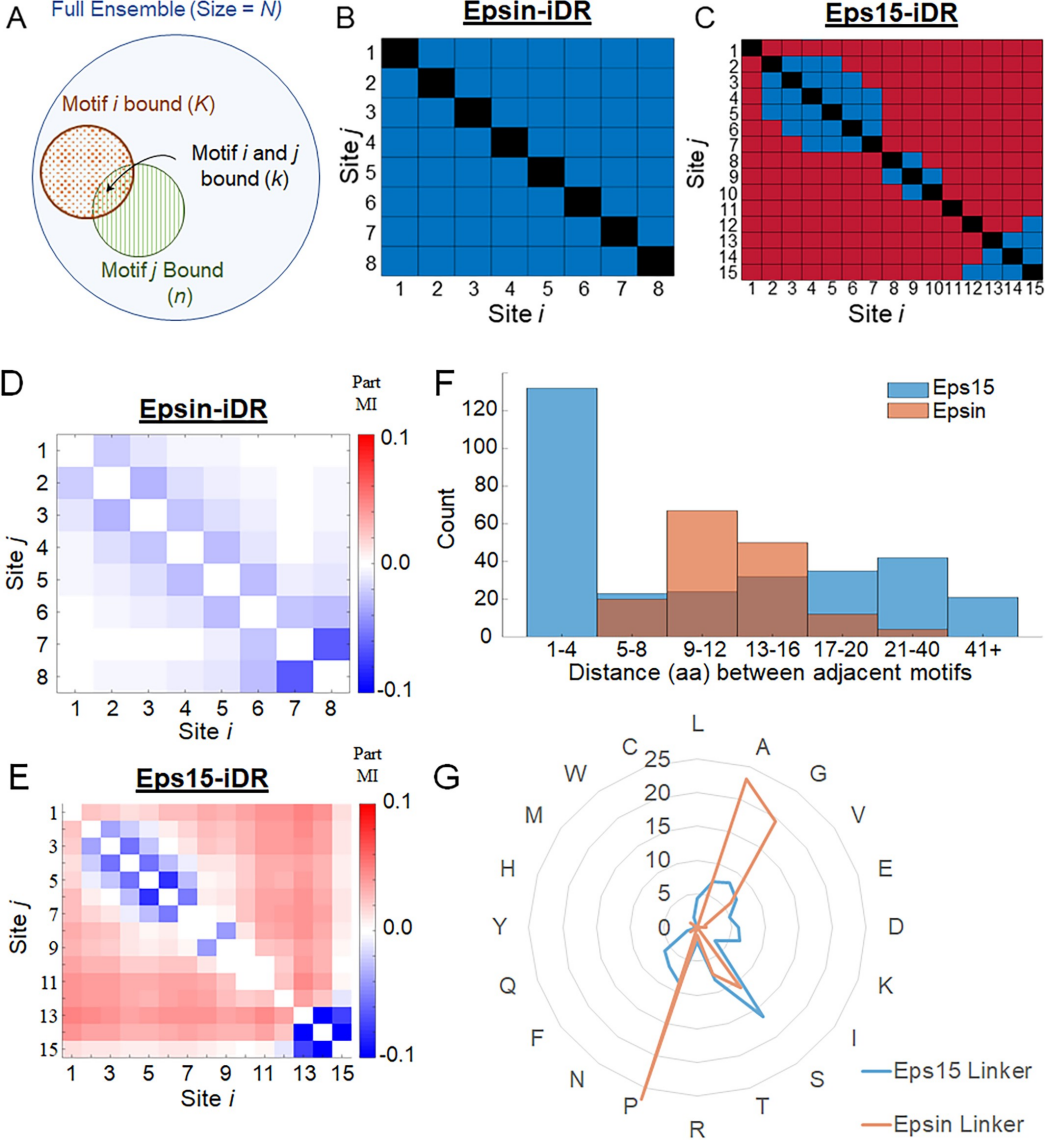
Eps15-iDR, but not Epsin-iDR, shows a statistical tendency toward co-accessibility of AP2α binding motifs. Schematic of the variables involved in the hypergeometric distribution to compute statistical co-accessibility. (B) Map showing whether the hypergeometric test shows independence (blue, p > 0.05) or a statistically significant tendency for co-accessibility (red, p < 0.05), for each pair of motifs (*i*, *j*) in the Epsin-iDR (C) same as B but for the Eps15-iDR. In the case of Eps15-iDR, multiple pairs of binding motifs were found to be statistically co-accessible. (D) Map showing partial mutual information (Part MI) for pairs of sites (*i*, *j*) in the Epsin-iDR. Positive values of Part MI (red) indicate that binding at *i* improves probability of binding at *j*. Negative values (blue) indicate that binding at *i* impairs binding at *j* and zero values (white) indicate no effect (E) same as D but for Eps15-iDR. (F) Histograms showing the distribution of inter-motif distances (i.e., distances between sequentially adjacent AP2α-binding motifs) in Epsin (Orange) and Eps15 (Blue) for all the species listed in S1 Text. Most neighboring motifs in Eps15 are within 1–4 residues of each other, while in Epsin, most are separated by 9–16 residues. (G) The amino acid composition of the sequences between adjacent binding motifs (linkers) in Epsin (orange) and Eps15 (blue). The linkers in Epsin are enriched for the amino acids Proline (27%), Alanine (23%) and Glycine (19.5%).

To shed light on how binding of one AP2α affects further capacity to bind in Epsin-iDR and Eps15-iDR, we measured whether the conformational accessibility of one binding motif is statistically correlated with the occupancy of another binding motif in the same conformer. A particular binding motif is considered to be *accessible* when docking-by-superposition of AP2α at that motif results in fewer than 100 VdW clashes (see methods). A pair of motifs is considered co-accessible if the ensembles permitting AP2α binding at one motif are disproportionately more likely to permit AP2α binding at the other motif. In other words, when one binding motif is occupied by AP2α, does that make it more likely for another binding motif in the same conformer to accommodate AP2α binding as well? To answer this question, we used two statistical metrics–the hypergeometric distribution, and mutual information. We first applied the hypergeometric test to subsets of Epsin-iDR/Eps15-iDR ensembles, with specific single or double-AP2α bound configurations. Given the size (number of conformers) of the unbound ensemble (*N*), the size of the ensemble allowing binding at motif *i* (*K*), and the size of the ensemble allowing binding at motif *j* (*n*), the hypergeometric test allows us to compute whether the observed number of structures binding AP2α at both motifs *i* and *j* simultaneously (*k*) is more than what would be expected if the two binding events were independent of each other. Pairs of motifs with the hypergeometric test p-values < 0.05 (after correction for multiple hypothesis testing) are considered to be co-accessible. Fig 4B and 4C shows which pairs of motifs exhibited statistical co-accessibility (red) or independence (blue) in Epsin-iDR and Eps15-iDR. Fig 4B shows that no pair of motifs in Epsin showed co-accessibility, whereas Fig 4C shows that many pairs of non-adjacent motifs in the Eps15-iDR showed statistical co-accessibility. The computed p-values appear in S8 Text. This suggests that AP2α binding at a motif may select for conformations that permit binding at a sequentially distant motif. Next, we used mutual information (MI) as a metric to obtain a measure of how much information is conveyed about binding capabilities at site *j* when we know the state of site *i*. We consider a pair of motifs to be interacting when MI between the sites in > 0 and the strength of the interaction depends on the value of MI. MI also suggests that there are only near-neighbor interactions in Epsin-iDR, whereas there are more interactions (between sequentially distant sites) in the Eps15-iDR (S9 Text). While MI gives a quantitative measure of the strength of interaction, it does not indicate if the non-independence is a positive correlation or an anti-correlation. Hence, we computed a metric called Partial Mutual Information (Part-MI, defined in S9 Text), which yields positive and negative sign, as well as strength. A positive interaction means that binding at site *i* increases the likelihood of binding at *j*, which might occur if two binding sites exhibit cooperativity. A negative interaction means that binding at site *i* decreases the likelihood of binding at *j*, which might occur if two binding sites are mutually exclusive due to steric clashes.

Part-MI calculations indicate that the interactions between sequentially adjacent binding sites are negative (hindering binding) in both Epsin-iDR and Eps15-iDR. In contrast, pairs of sites that are not sequentially adjacent show positive Part-MI values (positively affecting binding) in Eps15 but not in Epsin (Fig 4D and 4E). This is surprising because conformational correlations are not expected to persist very far along an excluded-volume polymer in the absence of electrostatic interactions or other structural organization. S10 Text shows the impact of AP2α binding on expansion of local regions (computed as the R_G_ of a 50-aa sliding window along the sequence); this computation confirms the existence of conformational correlations between non-sequential regions in our ensembles, but does not explain why this covariation occurs.

To understand the difference in conformational response to binding between the Eps15-iDR and the Epsin-iDR, we analyzed the spacing (number of residues along the sequence) and amino acid composition of the region between successive binding motifs. Fig 4F shows the distribution of these inter-motif distances (pooled for all species in S1 Text). The histogram shows that the inter-motif distances in Epsin are mostly in the range 5–20 (with the highest proportion in the range 9–16). In contrast, Eps15 has a very high proportion of motifs within 1–4 residues of each other. Eps15 also has a much higher proportion of long linkers (> 16aa) compared to Epsin. This bimodal distribution for Eps15 linker is conserved over evolution (S11 Text). More strikingly, analyzing the *H*. *sapiens* amino acid composition (Fig 4G) shows that the Epsin linker regions are 27% proline, which is a conformationally stiff reside, whereas the most abundant amino acid in Eps15 linker regions is the flexible polar reside Serine (16%).

## Discussion

Increased study of IDRs has shed light on their importance and function. While earlier IDR studies focused on folding-upon-binding and related mechanisms of order arising from disorder, more recent studies have suggested alternative modes of action where the disorder drives function. A typical example is the asymmetric molecular crowding of IDRs to generate forces and mechanical effects (e.g., Epsin [7]). However, unregulated crowding would be problematic for many reasons. For example, post-translational aggregation of IDRs could trigger the unfolded protein response. To harness molecular crowding for remodeling the plasma membrane during endocytosis, there would need to be regulation over space and time–namely, at the endocytic hotpot during vesicle formation. In this work, we use *in silico* methods of analysis to explore if *regulated* molecular crowding of the endocytic proteins Epsin and Eps15 might contribute to membrane deformation through *binding-induced expansion*.

To address this question, we first generated 3 million conformers for the intrinsically disordered regions of Epsin and Eps15 using TraDES, a Monte Carlo method that uses an excluded volume polymer model to generate sterically-feasible conformations. TraDES has been used previously to study ensembles of disordered regions in multiple contexts, including Wnt signaling and Actin elongation [46–48]. We then docked AP2α by superposition to the 8 DPW binding motifs in the Epsin-iDR and the 15 DPF motifs in the Eps15-iDR, and computed the dimensions and energetics of sub-ensembles of conformers that allowed different numbers of AP2α binding. Our first major result is that a larger fraction of the random Eps15-iDR conformers was capable of binding AP2α. Eps15-iDR was also capable of binding more copies of AP2α simultaneously, compared to the Epsin-iDR. Binding each additional copy of AP2α reduced the number of conformers in Epsin-iDR ensembles by 80–90% on average, whereas Eps15-iDR ensembles were reduced only by 20–66% (Tables 2 and 4). This could be because of the larger number of binding sites and/or fewer proline residues between binding sites of Eps15-iDR. Our second result is that Epsin-iDR ensembles that allowed more molecules of AP2α to bind exhibited longer lengths, suggesting they occupy larger steric volumes. This could be a result of the high proportion of prolines in the linker regions of Epsin, which makes the flanking regions less flexible. Eps15-iDR showed mild expansion in response to AP2α binding, for the first few AP2α molecules bound. However, binding additional copies of AP2α reversed this trend and brought the ensemble dimensions back to original values. Our third major result is that the binding of more molecules of AP2α selectively depleted the compact low-energy structures of Epsin-iDR. Taken together, these results suggest that AP2α binding causes a binding-induced expansion in the conformational space occupied by the energetically stable members of the Epsin-iDR ensemble. Given that the N-terminal region of Epsin can be anchored to the membrane at the endocytic hotspot, the ability of AP2α binding to induce expansion of the Epsin C-terminal IDR could contribute to increased molecular crowding and membrane deformation. In other words, our work adds the theoretical prediction of binding-induced crowding onto the Busch *et al*. [7] observation of crowding-induced membrane bending.

To complement our observation that the Eps15-iDR ensemble was more favorable than Epsin-iDR to bind multiple molecules of AP2α, we observed a tendency toward *co-accessibility* in Eps15-iDR (but not Epsin-iDR). This implies that the accessibility or occupancy of one Eps15-iDR binding motif could improve the accessibility of other binding motifs in the same conformer. Specifically, conformers in the Eps15-iDR 1-bound ensemble were found to have significantly greater-than-random tendency to accommodate binding of additional copies of AP2α simultaneously, although the co-accessible motifs tend to be non-sequential (shown by the blue diagonal and red off-diagonal in Fig 4C). This is a statistical argument based on ensembles that are so large that it cannot be coincidence of small number statistics. That fact does not prove that any effect is real, because our models are quite coarse, but it does indicate that performing additional runs of coarse modeling would not change this effect. Another view of this result is provided by the observation that conformers that allow binding at one site show local structural variations at other sequentially-distant parts of the conformer (S10 Text). If real, this would create a form of cooperativity between binding sites of Eps15, making it an ideal candidate to function as a recruiter of AP2α at the endocytic hotspot, since binding of AP2α at one motif would promote binding at other motifs. Future work with electrostatic modeling might be able to elucidate the structural mechanism for long-range correlations in the IDR structure.

The differing response of Epsin-iDR and Eps15-iDR to AP2α binding is curious given that the disordered regions of Epsin and Eps15 share many similarities–they bind to the same partner and have very similar sequence parameters such as the fraction of charged residues (FCR), net charge per residue (NCPR), and charge patterning (Kappa). Hence the differential response of the two iDRs to AP2α binding cannot solely be a function of the degree of disorder or high level sequence parameters, but has to depend on other features, for example, the number of binding sites, the interval between binding sites, or the amino acid composition. Hence, we looked at how individual binding motifs are distributed within the disordered regions. Across multiple species, the interval of polypeptide sequence between successive AP2α binding motifs in Eps15 (median = 8.4 ± 2.7 residues) was much shorter than in Epsin (median = 10.9 ± 1.4 residues) (S11 Text). In the human sequence, Epsin linkers had lengths of 7, 9, 10, 10, 14, 15 and 17, whereas Eps15 linkers were of lengths 2, 2, 2, 3, 3, 5, 10, 14, 17, 21, 25 and 58 (a combination of very short and long linkers as shown in Fig 4F). In Epsin, the smaller number of motifs and the greater length of sequence between motifs could allow AP2α binding at successive motifs simultaneously, provided the intervening sequence was sufficiently extended. In such a case, binding additional copies of AP2α would constrain multiple regions of the sequence toward extended conformations, especially given the high proportion of prolines in the linker regions (Fig 4G). This bias toward extended conformations provides a possible explanation for why binding-induced expansion might occur. However, in the case of Eps15, close spacing would make it far more difficult for Eps15 to bind AP2α at successive motifs. Another surprising observation was that there was strong non-independence in the AP2α accessibility of Eps15-iDR motifs, but not in Epsin-iDR motifs. The mechanism for Eps15 to exhibit non-independence between binding motifs (positive correlation between occupancy of sequentially distant binding sites) is not clear, but we do know that non-independence necessitates some interdependence or covariation in the conformational space, akin to allostery in folded proteins. While allostery seems highly improbable for an excluded volume polymer, there may be some long-distance effects of excluded volume resulting from AP2a disallowing certain conformations. The very tight evolutionary conservation of the Eps15 disordered region (even greater than the conservation of Epsin) implies that the structure-function relationship is intricately regulated, in ways we do not yet understand. A key overall question for experimental testing is whether the binding between Eps15 and AP2α exhibits cooperativity. Future experimental studies can test whether there is spatial proximity between the pairs of binding sites that showed co-accessibility in our studies. In addition, simulations of designed sequences having different lengths/spacing/composition between successive AP2α-binding motifs might confirm or refute contributing factors to the differences between Epsin and Eps15-iDRs to AP2α binding.

An important caveat of this study is that our modeling includes many first order approximations that create opportunities for error to be introduced and propagated. For example, our docking method (docking-by-superposition) involves rigid and static alignment of molecules, whereas true docking allows structures to change conformation according to the energetics of interaction. (Flexible docking is infeasible to perform for millions of conformers). In addition, docking by superposition uses specific crystal structures of AP2α bound to DPW and DPF peptides. Alternate structures for AP2α-peptide binding such as PDB structure 1KYD imply different orientations of binding and different dihedral angles for the peptide, which would change the number of atom clashes and the ensemble of feasible structures. Hence our results are dependent on the crystal structures used, as well as on the rigid approximations provided by docking-by-superposition. A final caveat is that we employ Monte-Carlo (MC) models of conformer generation to randomly sample the large conformational space. As a result, the generated conformers are all filtered for steric feasibility, but are not energetically minimized. While our model takes into account sequence constraints imposed by the bulkiness and excluded volume of each amino acid in sequence, it does not account for other factors such as non-covalent/energetic interactions between residues. Under physiological conditions, such energetic or environmental constraints could severely restrict the true conformational space available to these IDRs, resulting in low energy conformations that were missed by our conformational search. While techniques such as molecular dynamics simulations might capture these effects and produce energetically preferred conformations, they are computationally expensive or prohibitive for large ensembles. Therefore, in order to explore large ensembles, we have chosen to use MC models for a first-pass study that identifies qualitative changes in conformational ensembles and that prioritizes IDR hypotheses for further study. In other IDRs, TraDES-generated models [49] have been validated by experimentally-determined dimensions from SAXS. Hence, such methods are a reasonable method for generating novel hypotheses about IDR function, and for guiding the design of future studies that can use more focused and more accurate approaches, such as NMR, SAXS, or molecular dynamics.

In conclusion, we have used *de novo* methods of ensemble generation as a first step toward understanding the differences between the behavior of two disordered regions from proteins participating in Clathrin-mediated endocytosis. Subject to the approximations of our excluded-volume polymer model, our results show that while the two disordered regions share some sequence similarities, they exhibit different responses to partner binding. The mechanism of binding-induced expansion that we observe with the Epsin-iDR complements experimental results that show that the IDR of Epsin contributes to membrane deformation through molecular crowding. Binding-induced expansion could also be a general feature of other IDRs (both in CME and otherwise) and warrants deeper study. Future experimental studies of the Epsin-AP2α interaction can also help establish quantitative bounds for the steric pressure and membrane bending possible by these IDRs. Our work sheds light on a new means of regulating disorder and harnessing the thermodynamics of entropy towards carrying out the workload of cell biology.

## Methods

### Characterizing the disordered regions of Epsin and Eps15

The sequences of the human Epsin isoform 2 (Q9Y6I3-1) and Eps15 isoform 1 (P42566) were obtained from Uniprot. For both sequences, residue-specific propensities for disorder were predicted using the IUPred2A algorithm [50] for long disordered regions. Secondary structure propensities were predicted using JPred v4 [51]. For conservation analysis, Epsin and Eps15 sequences were used individually as inputs to BLASTp to obtain homologs, and diverse representatives were chosen manually for sequence alignment and display. Multiple Sequence Alignment (MSA) was performed using the Clustal Omega webserver and visualization was performed in JalView 2.11.0. The online tool CIDER v1.7 [43] was used to compute parameters such as Kappa that describe charge patterning of the sequence.

### Generation of conformational ensembles for the disordered regions of Epsin and Eps15

We defined the regions 232–471 from Epsin, and 498–830 from Eps15 as Epsin-iDR and Eps15-iDR respectively, since these regions were predicted to be disordered and contained all C-terminal binding motifs for the binding partner AP2α. Both Epsin-iDR and Eps15-iDR included a predicted helix region at the N-terminus, which was later used to superimpose generated conformers. The program FoldTraj from the TraDES package [33,39] was used to generate conformational ensembles of Epsin-iDR and Eps15-iDR with the following constraints. The dihedral angles (φ and ψ) for each of the binding motifs were constrained to values observed experimentally in structures of AP2α bound to either a DPW motif (PDB ID: 1KY6) or a DPF motif (PDB ID: 1KYF). In addition, the N-terminal residues in both sequences were restricted to adopt helical secondary structures. The TraDES program was run until an ensemble of 3 million conformers was generated for each Epsin-iDR and Eps15-iDR.

### Docking-by-superposition

Docking-by-superposition is a method of rigid docking that merges two protein structures—an existing crystal structure of a ligand-bound protein, and a new unbound ligand. As a result, this only involves moving the protein in space to its new ligand in the bound orientation, and does not allow for flexible adjustments in structure. In this work, we use docking-by-superposition to merge each DPW motif of Epsin-iDR with a DPW-containing peptide in the AP2α-bound PDB structure 1KY6, resulting in a pseudo-docking of the Epsin-iDR conformer with the AP2α crystal structure. The same is repeated for each DPF motif of Eps15-iDR and the DPF-containing peptide of PDB structure 1KYF. For each of the iDR conformers in the full ensembles, at each of its DP(W/F) motifs, the crystal structure of AP2α-DP(W/F) complex was docked on the conformer, such that the binding motif in the conformer and the same motif in the crystal structure were superimposed, using the *salign* module of TraDES. Note that the TraDES generation of conformers had already constrained the backbone of each motif to fit this crystal structure template. Docking-by-superposition resulted in a total of ~24 million dockings for Epsin (8 DPW motifs) and 45 million dockings for Eps15 (15 DPF motifs). In lieu of docking flexibility, we permit a limited number of hard-atom clashes, with the clash threshold estimated from the overall distribution of the number of clashes in each docking. A particular docking was then discarded as infeasible if it resulted in greater than 100 hard atomic Van der Waals clashes, as calculated using the *crashchk* module of TraDES. When a binding motif in a conformer had fewer than 100 clashes upon AP2α docking, it is considered an *accessible* motif. After discarding, every structure in the docked ensemble has AP2α bound at exactly one binding motif. We call these the 1-bound ensemble. Next, pairwise dockings were performed (for all pairs of motifs, for all structures in the 1-bound ensemble) and discarded if any of the three protein pairs (conformer-AP2α_1_, conformer-AP2α_2_, AP2α_1_-AP2α_2_) had more than 100 clashes. We call these filtered ensembles the 2-bound ensembles. All members of the 2-bound ensembles have AP2α bound at two binding motifs. Higher orders of AP2α dockings (3-bound, 4-bound ensembles etc.) were then inferred from these 1-bound and 2-bound ensembles (for example, a conformer was considered capable of binding AP2α at motifs 1, 3, and 5 simultaneously if the 2-bound ensemble contained instances of the same conformer bound to AP2α at motifs 1 and 3, motifs 3 and 5, and motifs 1 and 5).

### Statistical analysis of co-accessibility

The statistical dependence of the accessibility of a binding motif on the occupancy of other motifs in the same conformer was computed using a hypergeometric test and quantified using mutual information (MI). For either Epsin-iDR or Eps15-iDR, the hypergeometric test computes the expected size (number of conformers) of an ensemble that would allow binding at two distinct motifs *i* and *j* simultaneously (assuming binding at the motifs *i* and *j* are independent), using the sizes (number of conformers) of the following three ensembles as input: full ensemble, ensemble that allows AP2α binding at motif *i*, and ensemble that allows AP2α binding at motif *j*. For any pair of motifs *i* and *j* where the size of the observed ensemble is greater than expected, the test computes a p-value for statistical significance. Motif pairs with p < 0.05 (after BH correction for multiple hypothesis testing) were considered to show a statistical tendency toward co-accessibility. For any pair of motifs *i* and j, mutual information is a metric that quantifies the amount of information obtained about binding at motif *j* given knowledge about the state of motif *i*. The higher the value, the stronger the correlation between the occupancy of the sites. MI and another metric partial MI is computed as per S9 Text.

## Supporting information

S1 Text Multiple sequence alignment of Epsin IDR and Eps15 IDR. (PDF)

S2 Text Placement of the iDRs of Epsin and Eps15 in the phase plot of intrinsically disordered proteins. (PDF)

S3 Text Dimensions of the Epsin-iDR sub-ensembles for different numbers of AP2α binding and for alternate atom clash threshold values. (PDF)

S4 Text Proportions of Epsin-iDR conformers in different quadrants of the Energy-EED plots. (PDF)

S5 Text Dimensions of the Eps15-iDR sub-ensembles for different numbers of AP2α binding and for alternate atom clash threshold values. (PDF)

S6 Text Energy-EED plots for each sub-ensemble in Eps15. (PDF)

S7 Text Proportions of Eps15-iDR conformers in different quadrants of the Energy-EED plots. (PDF)

S8 Text p-values for Hypergeometric test of enrichment. (PDF)

S9 Text Mutual information between the occupancy at pairs of binding sites. (PDF)

S10 Text Sliding window R_G_. (PDF)

S11 Text Distances between sequentially adjacent AP2-binding motifs in Epsin and Eps15 across species. (PDF)
