# Supplementary material for: Computational modeling suggests binding-induced expansion of Epsin disordered regions upon association with AP2
Source: PLoS Comput Biol. 2021 Jan 6;17(1):e1008474. doi: 10.1371/journal.pcbi.1008474 (PMC7787433; doi:10.1371/journal.pcbi.1008474)
Supplement: S1 Text — (PDF) [file pcbi.1008474.s001.pdf]

S1.Multiple sequence alignment of Epsin-IDR and Eps15-IDR

S1.T1. List of species in the multiple sequence alignment from Figures 1C-D in the article

Organism	Common name
Homo sapiens	Human
Ciona intestinalis	Sea squirt (Vase tunicate)
Ixodes scapularis	Black-legged tick
Danio rerio	Zebra fish
Microcaecilia unicolor	Tiny Cayenne Caecilian
Xenopus tropicalis	Tropical clawed frog
Podarcis muralis	Common wall lizard
Protobothrops mucrosquamatus	Brown spotted pit viper
Gopherus evgoodei	Goodes thornscrub tortoise
Gallus gallus	Chicken
Ornithorhynchus anatinus	Platypus
Lagenorhynchus obliquidens	Pacific white-sided dolphin
Mus musculus	House mouse
Rattus norvegicus	Norway rat
Cricetulus griseus	Chinese hamster
Pteropus alecto	Black flying fox
Delphinapterus leucas	Beluga whale
Heterocephalus glaber	Naked mole-rat
Bos taurus	Cattle
Sus scrofa	Pig
Canis lupus familiaris	Dog
Pan troglodytes	Chimpanzee
Pongo abelii	Sumatran orangutan
Equus caballus	Horse

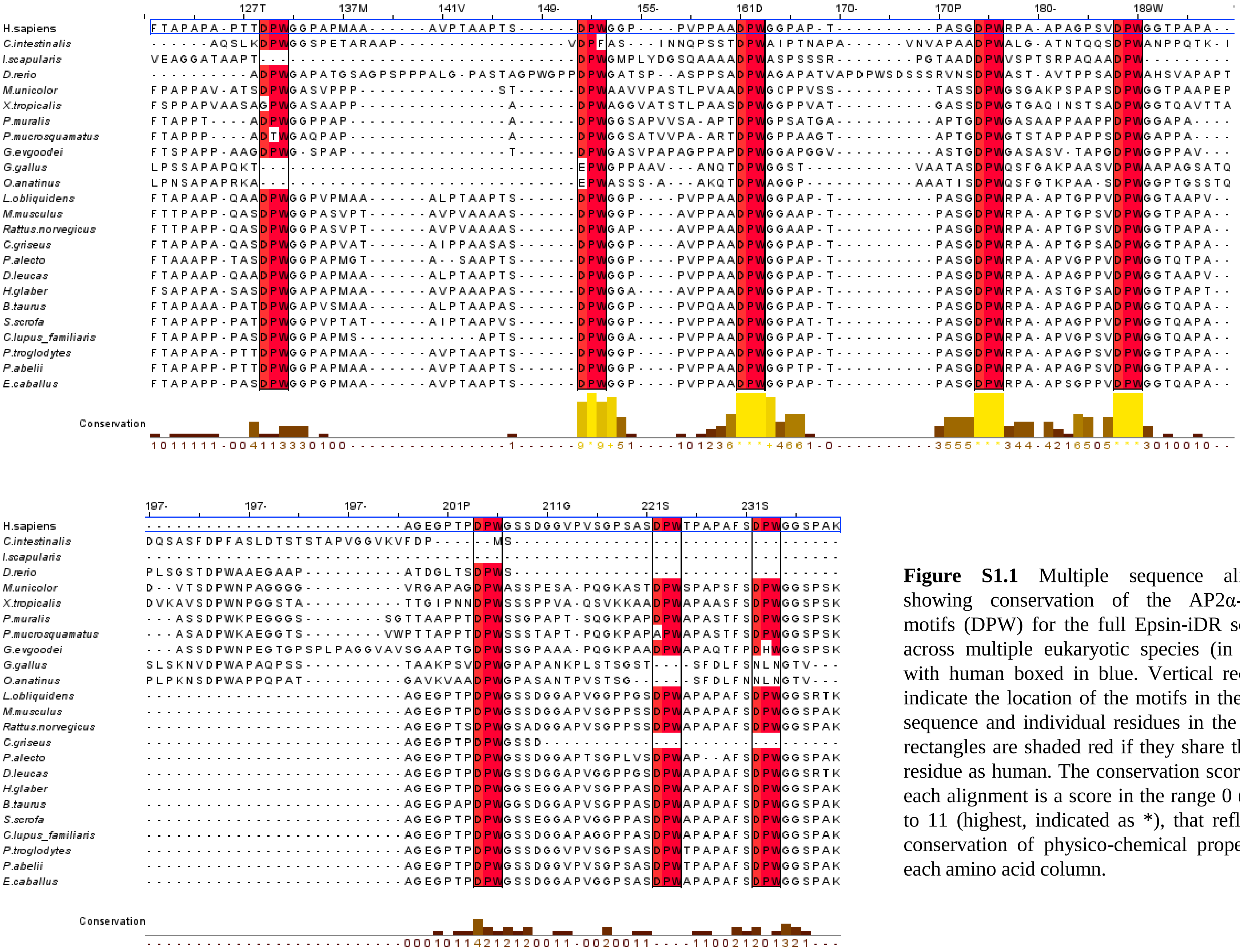

Figure S1.1 Multiple sequence alignment showing conservation of the AP2α-binding motifs (DPW) for the full Epsin-iDR sequence across multiple eukaryotic species (in S1.T1), with human boxed in blue. Vertical rectangles indicate the location of the motifs in the human sequence and individual residues in the vertical rectangles are shaded red if they share the same residue as human. The conservation score below each alignment is a score in the range 0 (lowest) to 11 (highest, indicated as *), that reflects the conservation of physico-chemical properties of each amino acid column.

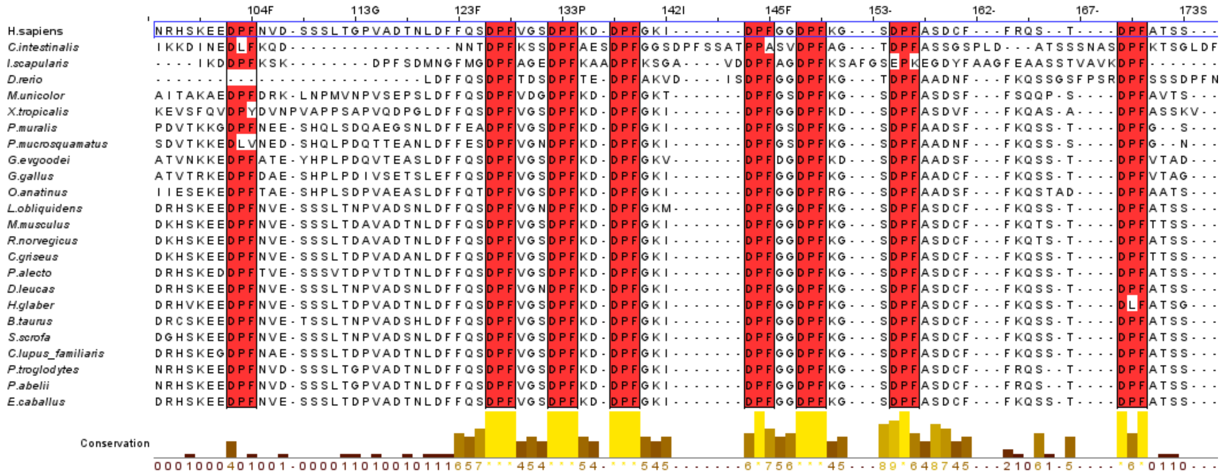

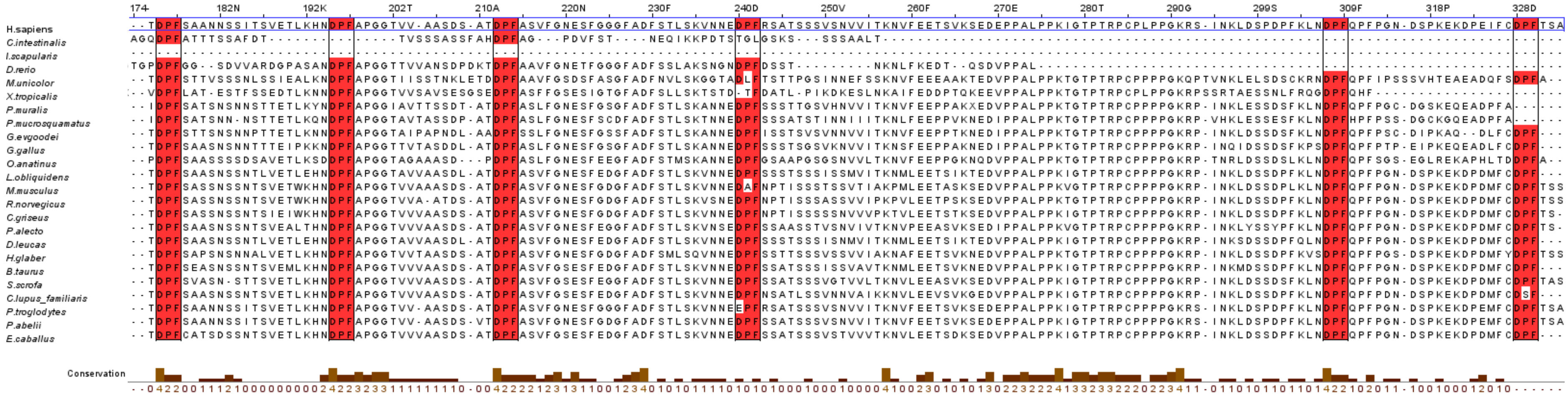

Figure S1.2 Multiple sequence alignment showing conservation of the AP2α-binding motifs (DPF) for the full Eps15-iDR sequence across multiple eukaryotic species (in S1.T1), with human boxed in blue. Vertical rectangles indicate the location of the motifs in the human sequence and individual residues in the vertical rectangles are shaded red if they share the same residue as human. The conservation score below each alignment is a score in the range 0 (lowest) to 11 (highest, indicated as *), that reflects the conservation of physico-chemical properties of each amino acid column.
