# Supplementary material for: Computational modeling suggests binding-induced expansion of Epsin disordered regions upon association with AP2
Source: PLoS Comput Biol. 2021 Jan 6;17(1):e1008474. doi: 10.1371/journal.pcbi.1008474 (PMC7787433; doi:10.1371/journal.pcbi.1008474)
Supplement: S2 Text — (PDF) [file pcbi.1008474.s002.pdf]

S2. Placement of the iDRs of Epsin and Eps15 in the Das *et. al.*, phase plot of intrinsically disordered proteins (1).

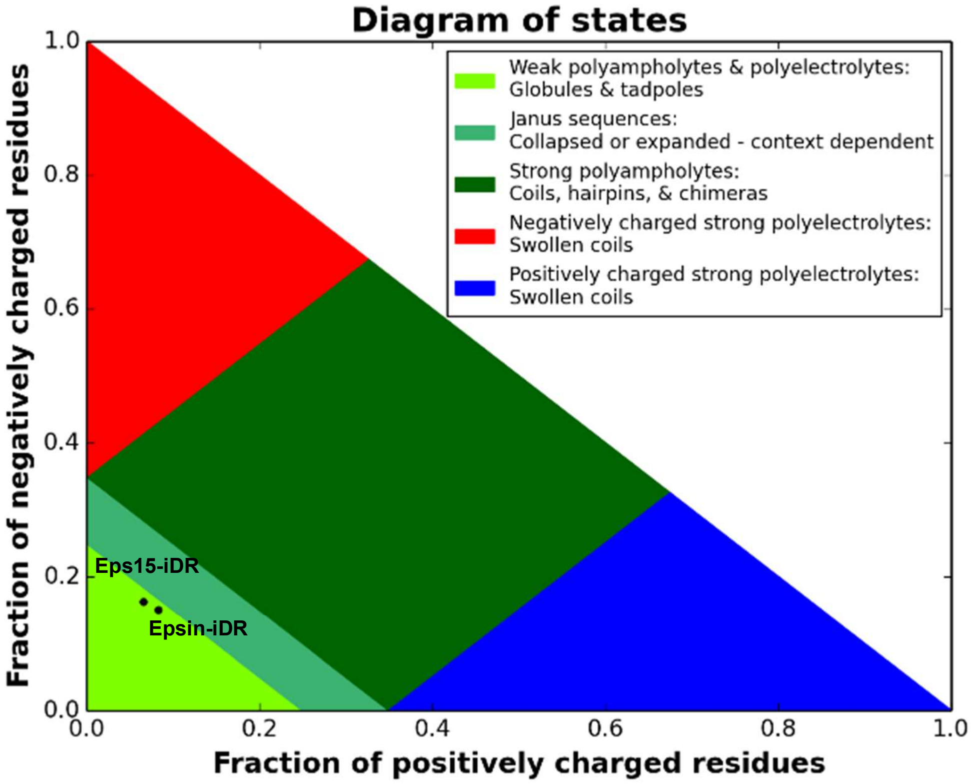

Figure S2.1. The disordered regions inspired from Epsin (Epsin-iDR) and Eps15 (Eps15-iDR) are at the phase boundary between globules/tadpoles and Janus sequences. IDRs can be classified into different phases depending on the proportion of positively charged and negatively charged residues in their sequence. Analyzing the sequences of the Epsin-iDR and Eps15-iDR show that they both localize to the boundary between two regions – the region for globular and tadpole like IDRs, and the region for Janus sequences (which can be collapsed or expanded depending on context).

REFERENCES

1. Das RK, Pappu RV. Conformations of intrinsically disordered proteins are influenced by linear sequence distributions of oppositely charged residues. Proc Natl Acad Sci USA. 2013 Aug 13;110(33):13392–7.
