# Supplementary material for: Computational modeling suggests binding-induced expansion of Epsin disordered regions upon association with AP2
Source: PLoS Comput Biol. 2021 Jan 6;17(1):e1008474. doi: 10.1371/journal.pcbi.1008474 (PMC7787433; doi:10.1371/journal.pcbi.1008474)
Supplement: S3 Text — (PDF) [file pcbi.1008474.s003.pdf]

S3. Dimensions of the Epsin-iDR sub-ensembles for different numbers of AP2α binding and for alternate atom clash threshold values

The main text (Table 2) contains the dimensions of Epsin-iDR sub-ensembles where sub-ensembles have been defined using an atom clash threshold of 100 to distinguish successful versus failed docking. In order to test whether the finding of binding-induced elongation depends on the choice of threshold for acceptable and unacceptable number of atom clashes, we re-computed the analysis using atom clash thresholds of 50 and 150 (S3.T1 and S3.T2, respectively).

Table S3.T1. Statistics of end-to-end distance (EED) and radius of gyration (R_G) for the Epsin-iDR ensembles that allow binding to increasing numbers of AP2α molecules. Atom clash threshold = 50

Ensemble	Total conformers	Average conformers	Std. dev conformers	Radius of Gyration (R_G)		End-to-end distance (EED)	
				Mean	Std. Dev	Mean	Std. Dev
Full ensemble	3,000,000	3,000,000.00	0.00	43.14	9.71	101.86	39.81
1-bound	1,644,899	282,647.25	78,914.15	44.03	9.76	103.88	40.11
2-bound	424,122	21,181.57	9,519.12	45.63	9.89	108.01	40.86
3-bound	51,985	1,233.57	754.02	47.93	10.10	114.35	42.35
4-bound	3,129	55.23	41.94	50.74	10.07	122.40	43.48

Table S3.T2. Statistics of end-to-end distance (EED) and radius of gyration (R_G) for the Epsin-iDR ensembles that allow binding to increasing numbers of AP2α molecules. Atom clash threshold = 150

Ensemble	Total conformers	Average conformers	Std. dev conformers	Radius of Gyration (R_G)		End-to-end distance (EED)	
				Mean	Std. Dev	Mean	Std. Dev
Full ensemble	3,000,000	3,000,000.00	0.00	43.14	9.71	101.86	39.81
1-bound	2,544,937	648,146.25	154,485.05	43.68	9.73	103.56	39.95
2-bound	1,431,694	116,613.32	40,590.90	45.02	9.82	107.60	40.42
3-bound	462,432	17,111.57	7,472.54	46.96	9.95	113.30	41.12
4-bound	79,559	2,015.20	1,021.65	49.34	10.05	120.30	41.79
5-bound	7,112	191.46	104.42	51.88	10.09	127.42	42.40
