# Supplementary material for: Computational modeling suggests binding-induced expansion of Epsin disordered regions upon association with AP2
Source: PLoS Comput Biol. 2021 Jan 6;17(1):e1008474. doi: 10.1371/journal.pcbi.1008474 (PMC7787433; doi:10.1371/journal.pcbi.1008474)
Supplement: S5 Text — (PDF) [file pcbi.1008474.s005.pdf]

S5. Dimensions of the Eps15-iDR sub-ensembles for different numbers of AP2α binding and for alternate atom clash threshold values

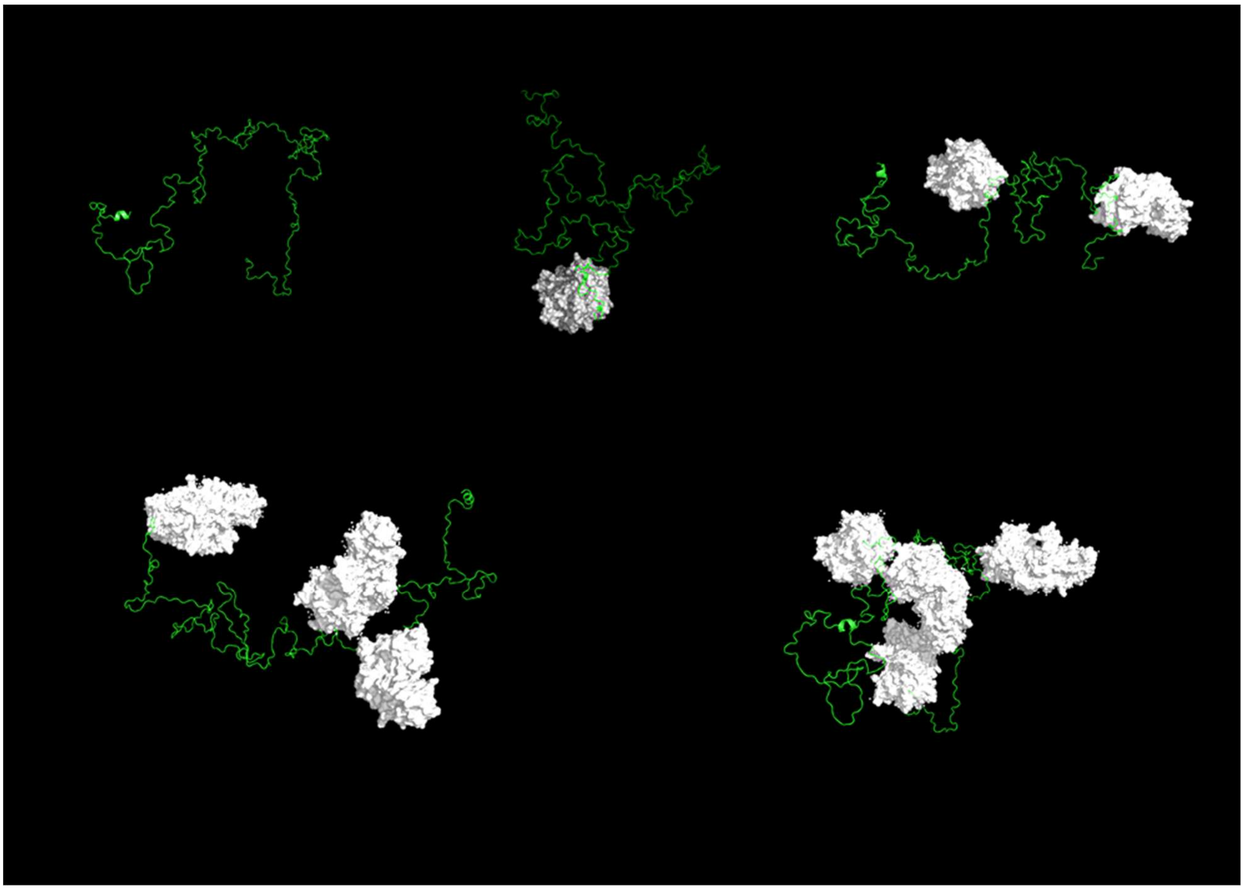

Figure S5.1. Examples of Eps15-iDR bound to AP2α. Examples of an unbound Eps15-iDR conformer and conformers that allow docking-by-superposition of 1, 2, 3 and 4 copies of AP2α.

Table 4 from the main text contains the dimensions of Eps15-iDR sub ensembles where sub-ensembles have been defined using an atom clash threshold of 100 to distinguish successful vs failed docking. In the following tables (S5.T1 and S5.T2), we provide results corresponding to atom clash thresholds of 50 and 150 respectively.

Table S5.T1. Statistics of end-to-end distance (EED) and radius of gyration (R_G) for the Eps15-iDR ensembles that allow binding to increasing numbers of AP2α molecules. Atom clash threshold = 50

Ensemble	Total conformers	Average conformers	Std. dev conformers	Radius of Gyration (R_G)		End-to-end distance (EED)	
				Mean	Std. Dev	Mean	Std. Dev
Full Ensemble	3,000,000	3,000,000.00	0.00	53.80	12.20	124.84	50.80
1-bound	2,947,872	824,100.60	220,297.83	53.88	12.20	125.10	50.81
2-bound	2,671,993	252,197.54	75,270.44	54.30	12.22	126.39	50.97
3-bound	2,063,157	96,971.93	24,785.68	55.20	12.33	129.06	51.43
4-bound	1,299,395	47,644.39	9,766.66	56.43	12.54	132.59	52.19
5-bound	685,381	27,561.43	4,867.74	57.35	12.84	135.21	53.03
6-bound	345,891	17,476.07	2,767.53	57.02	13.12	134.20	53.47
7-bound	198,326	11,807.70	1,662.45	55.49	12.93	129.89	52.62
8-bound	131,496	8,457.33	1,026.98	54.39	12.48	126.76	51.35
9-bound	88,960	6,422.77	648.91	54.04	12.29	125.76	50.94
10-bound	56,588	5,160.18	420.66	53.93	12.21	125.50	50.92

Table S5.T1. Statistics of end-to-end distance (EED) and radius of gyration (R_G) for the Eps15-iDR ensembles that allow binding to increasing numbers of AP2α molecules. Atom clash threshold = 150

Ensemble	Total conformers	Average conformers	Std. dev conformers	Radius of Gyration (R_G)		End-to-end distance (EED)	
				Mean	Std. Dev	Mean	Std. Dev
Full Ensemble	3,000,000	3,000,000.00	0.00	53.80	12.20	124.84	50.80
1-bound	2,994,052	1,138,332.60	274,965.31	53.81	12.20	124.88	50.79
2-bound	2,932,732	435,674.50	129,733.12	53.94	12.19	125.28	50.81
3-bound	2,691,104	180,343.89	52,924.47	54.39	12.20	126.67	50.94
4-bound	2,176,692	86,002.89	21,123.85	55.32	12.28	129.47	51.36

5-bound	1,480,188	48,109.24	9,192.51	56.67	12.47	133.41	52.13
6-bound	840,836	30,593.70	4,658.49	57.92	12.81	136.96	53.13
7-bound	425,725	21,183.47	2,691.40	58.16	13.25	137.55	54.01
8-bound	223,152	15,517.83	1,679.27	56.79	13.37	133.59	53.81
9-bound	133,626	11,858.28	1,086.94	55.04	12.87	128.54	52.30
10-bound	86,184	9,392.01	712.65	54.21	12.41	126.18	51.22
