# Supplementary material for: Computational modeling suggests binding-induced expansion of Epsin disordered regions upon association with AP2
Source: PLoS Comput Biol. 2021 Jan 6;17(1):e1008474. doi: 10.1371/journal.pcbi.1008474 (PMC7787433; doi:10.1371/journal.pcbi.1008474)
Supplement: S6 Text — (PDF) [file pcbi.1008474.s006.pdf]

S6. Energy-EED plots for each sub-ensemble in Eps15

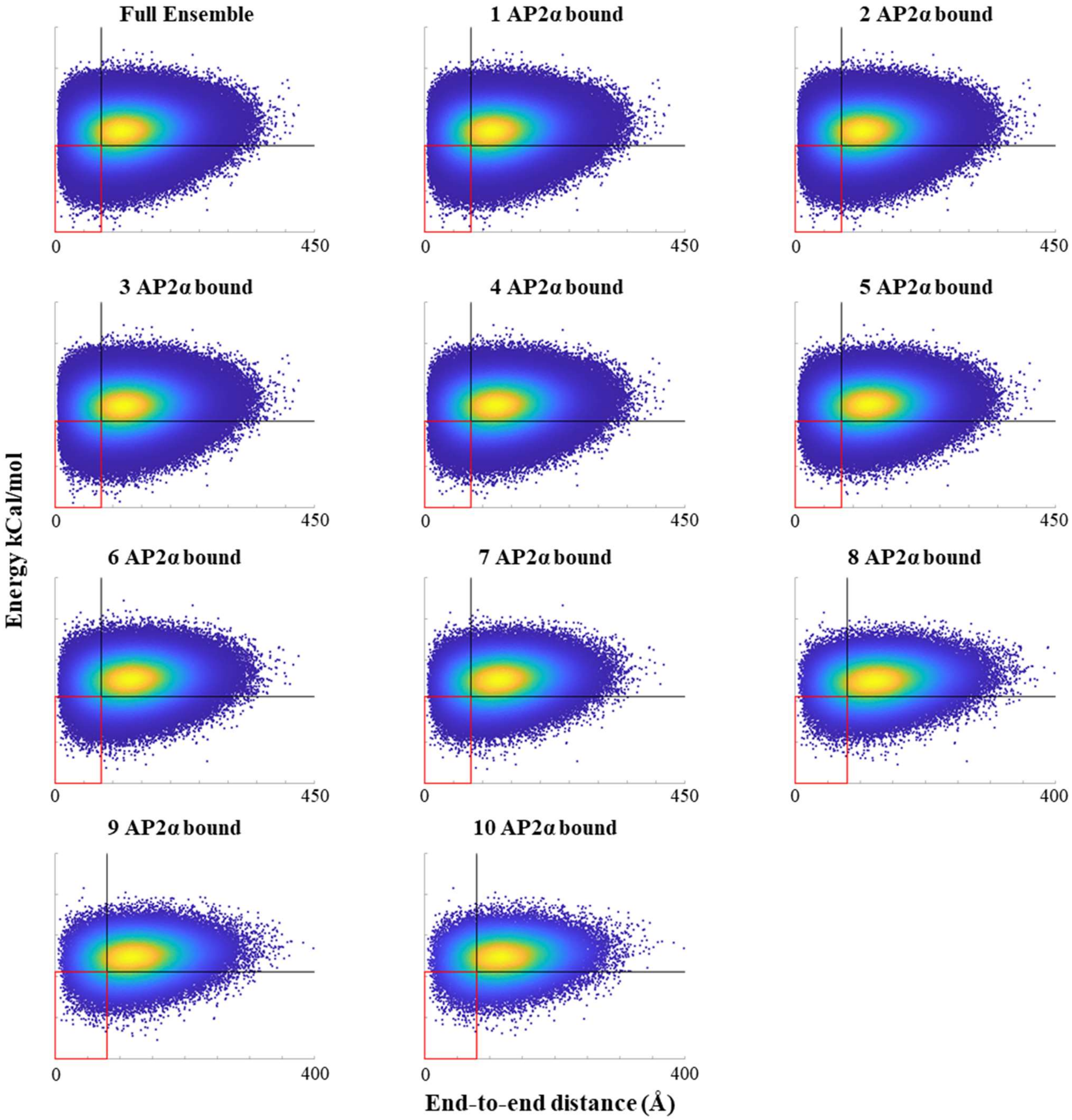

Figure S6.1 Density scatter plots showing the distribution of conformers in energy-EED space for different sub-ensembles of Eps15. Within each plot, the vertical and horizontal black lines represent the 20th percentile thresholds for low/high EED and low/high energy, respectively. The red rectangle at the lower left of each plot represents the group of compact low-energy conformers.
