# Supplementary material for: Computational modeling suggests binding-induced expansion of Epsin disordered regions upon association with AP2
Source: PLoS Comput Biol. 2021 Jan 6;17(1):e1008474. doi: 10.1371/journal.pcbi.1008474 (PMC7787433; doi:10.1371/journal.pcbi.1008474)
Supplement: S7 Text — (PDF) [file pcbi.1008474.s007.pdf]

S7. Proportions of Eps15-iDR conformers in different quadrants of the Energy-EED plots

For each Eps15-iDR sub-ensemble (1-bound, 2-bound etc.), Table 5 from the main text shows the relative proportions of Eps15-iDR conformers in different quadrants of the Energy-EED plot, when using an atom clash threshold of 100. In order to check if the observed differences between sub ensembles could be a result of random chance, we ran the following simulations for each sub-ensemble. If N is the number of conformers in the sub-ensemble, we repeatedly picked N conformers from the full ensemble at random, and for each set of N conformers, we used the same energy and EED thresholds as in Table 5 to sort them into different quadrants. We then quantified the proportion of conformers in each quadrant. This process was repeated 1000 times per sub ensemble and the table below shows the mean and standard deviation of the 1000 random samples. It can be seen that in the random samples, the proportions are tightly clustered around the proportions observed with the full ensemble, whereas the actual proportions observed in Table 5 show larger sub-ensemble specific differences.

Table S7.T1 Relative fractions of Eps15-iDR conformers in different regions of the Energy–EED state space. Elements of the table represent mean and standard deviations obtained using 1000 random samples of the same size (conformer number) as the corresponding sub-ensemble. Thresholds for EED and Energy were set at the 20th percentile of the corresponding values in the full ensemble. Atom clash threshold = 100.

Ensemble	Low EED<br>Low Energy	Low EED<br>High Energy	High EED<br>High Energy	High EED<br>Low Energy
Full ensemble	0.05	0.15	0.65	0.15
1-bound	0.05 +/- 8.4e-06	0.15 +/- 1.4e-05	0.65 +/- 1.9e-05	0.15 +/- 1.4e-05
2-bound	0.05 +/- 2.7e-05	0.15 +/- 4.3e-05	0.65 +/- 5.8e-05	0.15 +/- 4.3e-05
3-bound	0.05 +/- 5.7e-05	0.15 +/- 9e-05	0.65 +/- 0.00012	0.15 +/- 9.2e-05
4-bound	0.05 +/- 9.4e-05	0.15 +/- 0.00016	0.65 +/- 0.00021	0.15 +/- 0.00016
5-bound	0.05 +/- 0.00016	0.15 +/- 0.00026	0.65 +/- 0.00035	0.15 +/- 0.00026
6-bound	0.05 +/- 0.00025	0.15 +/- 0.00038	0.65 +/- 0.00053	0.15 +/- 0.00041

7-bound	0.05 +/- 0.00038	0.15 +/- 0.00061	0.65 +/- 0.00079	0.15 +/- 0.0006
8-bound	0.05 +/- 0.0005	0.15 +/- 0.00085	0.65 +/- 0.0011	0.15 +/- 0.00083
9-bound	0.05 +/- 0.00064	0.15 +/- 0.001	0.65 +/- 0.0014	0.15 +/- 0.0011
10-bound	0.05 +/- 0.0008	0.15 +/- 0.0013	0.65 +/- 0.0017	0.15 +/- 0.0013

The analysis in Tables 5 (main text) and S7.T1 were repeated using different values for atom clash thresholds. The results can be seen in the tables S7.T2 and S7.T3. For conciseness we indicate both the true fractions of conformers and the values obtained using random sampling in the same table.

Table S7.T2 Relative proportions (in fractions) of Eps15-iDR conformers in different regions of the Energy – EED (end-to-end distance) state space. Thresholds for EED and Energy were set at the 20th percentile of the corresponding values in the full ensemble. True values (values from sub ensembles obtained through docking) are in bold. Note there is slight trend toward elongation of Eps15 when accommodating two to five copies of the AP2α molecule, and this trend is reversed for more than 5 copies of AP2α bound. Values in parentheses represent mean and standard deviations obtained using 1000 random samples of the same size (conformer number) as the corresponding sub-ensemble. Atom clash threshold = 50.

Ensemble	Low EED<br>Low Energy	Low EED<br>High Energy	High EED<br>High Energy	High EED<br>Low Energy
Full ensemble	**0.05**	**0.15**	**0.65**	**0.15**
1-bound	**0.05**<br>(0.05 +/- 1.6e-05)	**0.15**<br>(0.15 +/- 2.7e-05)	**0.65**<br>(0.65 +/- 3.4e-05)	**0.15**<br>(0.15 +/- 2.6e-05)
2-bound	**0.05**<br>(0.05 +/- 4.5e-05)	**0.15**<br>(0.15 +/- 7.4e-05)	**0.66**<br>(0.65 +/- 9.7e-05)	**0.15**<br>(0.15 +/- 7.1e-05)
3-bound	**0.04**<br>(0.05 +/- 8.4e-05)	**0.14**<br>(0.15 +/- 0.00014)	**0.68**<br>(0.65 +/- 0.00019)	**0.14**<br>(0.15 +/- 0.00014)
4-bound	**0.04**<br>(0.05 +/- 0.00014)	**0.13**<br>(0.15 +/- 0.00024)	**0.7**<br>(0.65 +/- 0.00032)	**0.13**<br>(0.15 +/- 0.00023)
5-bound	**0.03**<br>(0.05 +/- 0.00023)	**0.12**<br>(0.15 +/- 0.00039)	**0.72**<br>(0.65 +/- 0.0005)	**0.13**<br>(0.15 +/- 0.00038)
6-bound	**0.04**<br>(0.05 +/- 0.00034)	**0.13**<br>(0.15 +/- 0.00056)	**0.71**<br>(0.65 +/- 0.00078)	**0.13**<br>(0.15 +/- 0.00058)
7-bound	**0.04**<br>(0.05 +/- 0.00048)	**0.14**<br>(0.15 +/- 0.00074)	**0.68**<br>(0.65 +/- 0.001)	**0.14**<br>(0.15 +/- 0.00078)

8-bound	**0.05**<br>(0.05 +/- 0.00058)	**0.15**<br>(0.15 +/- 0.00096)	**0.66**<br>(0.65 +/- 0.0013)	**0.15**<br>(0.15 +/- 0.00097)
9-bound	**0.05**<br>(0.05 +/- 0.00071)	**0.15**<br>(0.15 +/- 0.0011)	**0.66**<br>(0.65 +/- 0.0015)	**0.15**<br>(0.15 +/- 0.0012)
10-bound	**0.05**<br>(0.05 +/- 0.0009)	**0.15**<br>(0.15 +/- 0.0015)	**0.66**<br>(0.65 +/- 0.0019)	**0.15**<br>(0.15 +/- 0.0015)

Table S7.T3 Relative proportions (in fractions) of Eps15-iDR conformers in different regions of the Energy – EED (end-to-end distance) state space. Thresholds for EED and Energy were set at the 20th percentile of the corresponding values in the full ensemble. True values (values from sub ensembles obtained through docking) are in bold. Note there is slight trend toward elongation of Eps15 when accommodating two to five copies of the AP2α molecule, and this trend is reversed for more than 5 copies of AP2α bound. Values in parentheses represent mean and standard deviations obtained using 1000 random samples of the same size (conformer number) as the corresponding sub-ensemble. Atom clash threshold = 150.

Ensemble	Low EED<br>Low Energy	Low EED<br>High Energy	High EED<br>High Energy	High EED<br>Low Energy
Full ensemble	**0.05**	**0.15**	**0.65**	**0.15**
1-bound	**0.049**<br>(0.05 +/- 5.6e-06)	**0.15**<br>(0.15 +/- 8.9e-06)	**0.65**<br>(0.65 +/- 1.2e-05)	**0.15**<br>(0.15 +/- 9e-06)
2-bound	**0.048**<br>(0.05 +/- 1.9e-05)	**0.149**<br>(0.15 +/- 3e-05)	**0.653**<br>(0.65 +/- 4.3e-05)	**0.15**<br>(0.15 +/- 3.2e-05)
3-bound	**0.045**<br>(0.05 +/- 4.3e-05)	**0.144**<br>(0.15 +/- 7e-05)	**0.663**<br>(0.65 +/- 9.4e-05)	**0.148**<br>(0.15 +/- 6.9e-05)
4-bound	**0.04**<br>(0.05 +/- 7.8e-05)	**0.136**<br>(0.15 +/- 0.00013)	**0.681**<br>(0.65 +/- 0.00017)	**0.143**<br>(0.15 +/- 0.00012)
5-bound	**0.034**<br>(0.05 +/- 0.00012)	**0.125**<br>(0.15 +/- 0.00022)	**0.705**<br>(0.65 +/- 0.00027)	**0.136**<br>(0.15 +/- 0.0002)
6-bound	**0.031**<br>(0.05 +/- 0.0002)	**0.116**<br>(0.15 +/- 0.00033)	**0.724**<br>(0.65 +/- 0.00043)	**0.129**<br>(0.15 +/- 0.00033)
7-bound	**0.032**<br>(0.05 +/- 0.00031)	**0.117**<br>(0.15 +/- 0.00051)	**0.723**<br>(0.65 +/- 0.00068)	**0.129**<br>(0.15 +/- 0.00049)
8-bound	**0.038**<br>(0.05 +/- 0.00045)	**0.128**<br>(0.15 +/- 0.00073)	**0.699**<br>(0.65 +/- 0.00097)	**0.135**<br>(0.15 +/- 0.00074)
9-bound	**0.044**<br>(0.05 +/- 0.00056)	**0.141**<br>(0.15 +/- 0.00094)	**0.67**<br>(0.65 +/- 0.0013)	**0.144**<br>(0.15 +/- 0.00096)
10-bound	**0.048**<br>(0.05 +/- 0.00075)	**0.147**<br>(0.15 +/- 0.0012)	**0.659**<br>(0.65 +/- 0.0016)	**0.147**<br>(0.15 +/- 0.0012)
