# Supplementary material for: Computational modeling suggests binding-induced expansion of Epsin disordered regions upon association with AP2
Source: PLoS Comput Biol. 2021 Jan 6;17(1):e1008474. doi: 10.1371/journal.pcbi.1008474 (PMC7787433; doi:10.1371/journal.pcbi.1008474)
Supplement: S8 Text — (PDF) [file pcbi.1008474.s008.pdf]

S8. p-values for Hypergeometric test of enrichment

Hypergeometric distribution describes the number of successes that can be expected in a sample given the total number of successes in a population. The test is used to verify if the sample is enriched for successes by computing the probability of seeing as many successes in a random sample of the same size as the chosen sample. In our case, the overall population is the full ensemble of 3 million conformers, and "successes" are defined as the conformers that can bind at site i. The chosen sample is the set of conformers that can bind at site j. Thus, the hypergeometric test is used to compute the probability of seeing as many conformers binding at sites i in a random sample of the size of the chosen sample (conformers binding at site j). When the probability is less than 0.05, we say that there is enrichment of conformers binding at site i in the set binding at site j. In such a case, we conclude that binding at sites i and j are not independent of each other.

Figures 4B and 4C in the main text show heatmaps of hypergeometric p-values that were computed as follows. For both Epsin-iDR and Eps15-iDR, uncorrected p-values were first obtained from the hypergeometric distribution using the sizes (number of conformers) of the following four ensembles as input.

(1) full ensemble (N)
(2) ensemble allowing binding at motif i (K)
(3) ensemble allowing binding at motif j (n), and
(4) ensemble allowing simultaneous binding at motifs i and j (k),

p-values were computed as the hypergeometric probability that ensemble 4 (simultaneous binding at motifs i and j), would at least that many structures (k), if binding at motifs i and j are indeed independent events.

$$p = P(X \geq k), where\ X \sim Hypergeometric(N, K, n).$$

The computed p-values were then corrected for multiple hypothesis testing using the Benjamini-Hochberg method. Corrected p-values for different values of atom clash thresholds (50,100,150) can be found in S8.Tables 2-4 (Epsin-iDR) and S8.Tables 6-8 (Eps15-iDR). Figures 4B and C correspond to the corrected p-values obtained using atom clash threshold = 100.

S8.Table1 – Number of conformers in 1-bound and 2-bound Epsin-iDR conformers for different values of Atom clash thresholds

Site 1	Site 2	<150	<100	<50
**1-bound ensemble**				
1	NA	596022	481437	270071
2	NA	568815	456690	256962
3	NA	552385	442728	247747
4	NA	515953	399394	191931
5	NA	610149	491307	278208

6	NA	644835	516438	284521
7	NA	690928	543246	267249
8	NA	1006083	815639	464489
**2-bound ensemble**				
1	2	81557	51606	15596
1	3	94336	60228	18676
1	4	96705	59667	15005
1	5	115739	74705	23636
1	6	126049	81087	25020
1	7	137651	87275	23074
1	8	196573	128770	41736
2	3	51797	31743	8917
2	4	76927	46719	11321
2	5	101278	64842	20501
2	6	115168	73416	22809
2	7	129376	81344	21748
2	8	184600	119935	39165
3	4	53053	31338	6999
3	5	87109	55457	16911
3	6	107644	68523	20619
3	7	124292	78295	20648
3	8	177022	114407	36626
4	5	54204	31963	7170
4	6	92928	56568	13798
4	7	115798	70502	15745
4	8	163161	101373	26221
5	6	83584	52408	15525
5	7	125433	79034	20898
5	8	185191	120520	38793
6	7	106324	66066	16421
6	8	173749	112411	34625
7	8	107925	65152	14881

S8.Table2 – p-values for co-accessibility between pairs of binding motifs in the Epsin-iDR. Atom clash threshold = 100

MOTIF	1	2	3	4	5	6	7	8
**1**	NA							
**2**	1	NA						
**3**	1	1	NA					
**4**	1	1	1	NA				

**5**	1	1	1	1	NA			
**6**	1	1	1	1	1	NA		
**7**	1	1	1	1	1	1	NA	
**8**	1	1	1	1	1	1	1	NA

S8.Table3 – p-values for co-accessibility between pairs of binding motifs in the Epsin-iDR. Atom clash threshold = 50

MOTIF	1	2	3	4	5	6	7	8
**1**	NA							
**2**	1	NA						
**3**	1	1	NA					
**4**	1	1	1	NA				
**5**	1	1	1	1	NA			
**6**	1	1	1	1	1	NA		
**7**	1	1	1	1	1	1	NA	
**8**	1	1	1	1	1	1	1	NA

S8.Table4 – p-values for co-accessibility between pairs of binding motifs in the Epsin-iDR. Atom clash threshold = 150

MOTIF	1	2	3	4	5	6	7	8
**1**	NA							
**2**	1	NA						
**3**	1	1	NA					
**4**	1	1	1	NA				
**5**	1	1	1	1	NA			
**6**	1	1	1	1	1	NA		
**7**	1	1	1	1	1	1	NA	
**8**	1	1	1	1	1	1	1	NA

S8.Table5 – Number of conformers in 1-bound and 2-bound Eps15-iDR conformers for different values of Atom clash thresholds

Site 1	Site 2	<150	<100	<50
**1-bound ensemble**				
1	NA	1103958	976027	780143
2	NA	933234	822585	658422
3	NA	924550	806327	651733
4	NA	1087120	963314	766419
5	NA	1117720	982303	787526

6	NA	963241	846651	672377
7	NA	963480	842505	675663
8	NA	1004881	888449	725898
9	NA	1144410	1025769	851611
10	NA	1133306	1012078	821424
11	NA	955421	841305	690057
12	NA	1136649	1016473	843388
13	NA	1162240	1042788	857970
14	NA	1429894	1289242	1057436
15	NA	2014885	1900792	1521442
**2-bound ensemble**				
1	2	356276	290112	207385
1	3	353329	283993	201898
1	4	404596	326692	225318
1	5	420718	336334	232758
1	6	374097	300957	209748
1	7	376677	302151	212669
1	8	395937	320858	228459
1	9	445755	363465	258642
1	10	446783	363963	256067
1	11	390275	316623	228396
1	12	456084	373941	270447
1	13	471810	388574	281559
1	14	561344	461088	327495
1	15	750622	633074	427609
2	3	226724	181732	134225
2	4	300634	241631	168415
2	5	327134	261957	182419
2	6	299866	241102	168996
2	7	308831	247813	175043
2	8	332785	269123	192246
2	9	374341	305489	218301
2	10	378455	307910	217541
2	11	333451	270418	195753
2	12	388954	318158	230603
2	13	401020	329612	239134
2	14	476260	390007	277000
2	15	634826	533718	360045
3	4	247713	198301	140567
3	5	303546	240521	169456
3	6	284443	226431	159866
3	7	298395	237414	168580
3	8	325745	261086	187578

3	9	367507	296163	212972
3	10	373895	301082	214168
3	11	329623	265161	192753
3	12	384859	311920	227050
3	13	397198	323419	235278
3	14	470798	381466	272550
3	15	626784	521248	354900
4	5	320932	256014	176461
4	6	303146	242499	166692
4	7	327752	261948	181397
4	8	368297	297255	208041
4	9	418318	340370	239294
4	10	427118	346697	240372
4	11	374103	301947	214137
4	12	439754	359066	255099
4	13	454109	371968	263471
4	14	543373	444145	308947
4	15	732451	617466	409499
5	6	232744	183585	129419
5	7	310919	246669	172434
5	8	370025	296417	209065
5	9	424125	342854	242534
5	10	434939	351159	244661
5	11	383027	307397	218716
5	12	449924	364126	260123
5	13	466817	379515	269758
5	14	556932	451137	315259
5	15	752299	629325	419999
6	7	229371	180486	128191
6	8	320811	258374	183365
6	9	369060	299371	212599
6	10	381039	308949	216779
6	11	338646	273537	196489
6	12	396875	323049	231779
6	13	411374	336272	240774
6	14	488281	398038	279200
6	15	653004	547129	365080
7	8	311486	250429	179924
7	9	363743	293886	210681
7	10	380743	307499	217570
7	11	338453	272889	197634
7	12	397685	323207	234202
7	13	412113	335866	242421

7	14	490191	398198	282108
7	15	653529	545479	368340
8	9	321624	260529	188876
8	10	383189	312113	223721
8	11	345764	281022	206060
8	12	409640	335821	246286
8	13	426715	350732	256477
8	14	507266	415721	298364
8	15	678952	572319	392422
9	10	418914	344025	248145
9	11	379829	310669	228652
9	12	455249	375618	277050
9	13	475509	393901	289148
9	14	569788	471323	340514
9	15	767322	654482	451504
10	11	349815	285682	208536
10	12	443625	365177	265665
10	13	471072	389667	282441
10	14	563318	465226	331345
10	15	757302	644274	437049
11	12	374490	307633	229391
11	13	409261	337996	251387
11	14	483936	397211	289841
11	15	640653	538609	372424
12	13	467937	389568	290216
12	14	555779	460884	336344
12	15	743547	632831	439106
13	14	418057	336713	237846
13	15	683357	580929	395311
14	15	818314	698828	470218

S8.Table 6– p-values for co-accessibility between pairs of binding motifs in the Eps15-iDR. Atom Clash threshold = 100

MOTIF	1	2	3	4	5	6	7	8	9	10	11	12	13	14	15
**1**	NA														
**2**	0	NA													
**3**	0	1	NA												
**4**	1.90E -268	1	1	NA											

**5**	0	1	1	1	NA										
**6**	0	1.16E-145	1	1	1	NA									
**7**	0	0	1.61E-220	1	1	1	NA								
**8**	0	0	0	1.01E-229	6.08E-50	5.54E-102	0.005777	NA							
**9**	0	0	0	3.63E-180	2.43E-73	5.99E-157	6.11E-56	1	NA						
**10**	0	0	0	0	0	0	0	7.39E-240	1	NA					
**11**	0	0	0	0	0	0	0	0	0	2.66E-07	NA				
**12**	0	0	0	0	0	0	0	0	0	0	0	NA			
**13**	0	0	0	0	0	0	0	0	0	0	0	0	NA		
**14**	0	0	0	0	0	0	0	0	0	0	0	0	1	NA	
**15**	0.00E+00	2.37E-249	2.89E-173	1.02E-74	1.52E-70	6.23E-179	2.21E-213	7.43E-135	6.83E-31	1.10E-14	4.97E-50	1	1	1	NA

<u>S8.Table 7– p-values for co-accessibility between pairs of binding motifs in the Eps15-iDR. Atom Clash threshold = 50</u>

MOTIF	1	2	3	4	5	6	7	8	9	10	11	12	13	14	15
**1**	NA														
**2**	0	NA													
**3**	0	1	NA												
**4**	0	0.288	1	NA											
**5**	0	4.51E-201	1	1	NA										
**6**	0	0	0	1	1	NA									
**7**	0	0	0	1.88E-169	1	1	NA								
**8**	0	0	0	0	0	0	0	NA							
**9**	0	0	0	0	0	0	0	1	NA						
**10**	0	0	0	0	0	0	0	0	0	NA					
**11**	0	0	0	0	0	0	0	0	0	0	NA				
**12**	0	0	0	0	0	0	0	0	0	0	0	NA			
**13**	0	0	0	0	0	0	0	0	0	0	0	0	NA		
**14**	0	0	0	0	0	0	0	0	0	0	0	0	1	NA	
**15**	0	0	0	0	0	0	0	0	0	0	0	2.91E-188	1	1	NA

S8.Table 8 – p-values for co-accessibility between pairs of binding motifs in the Eps15-iDR. Atom Clash threshold = 150

MOTIF	1	2	3	4	5	6	7	8	9	10	11	12	13	14	15
**1**	NA														
**2**	7.46E-242	NA													
**3**	1.87E-252	1	NA												
**4**	7.11E-30	1	1	NA											
**5**	4.46E-120	1	1	1	NA										
**6**	0	0.372	1	1	1	NA									
**7**	0	1.42E-130	5.91E-05	1	1	1	NA								
**8**	0	0	0	3.14E-26	1	1	1	NA							
**9**	0	0	1.728e-317	2.96E-19	1	2.85E-05	1	1	NA						
**10**	0	0	0	0	1.71E-214	0	0	1.38E-19	1	NA					
**11**	0	0	0	0	0	0	0	0	0	1	NA				
**12**	0	0	0	0	0	0	0	0	0	4.30E-267	3.46E-223	NA			
**13**	0	0	0	0	0	0	0	0	0	0	0	0	NA		
**14**	0	0	0	0	0	0	0	0	0	0	0	1.47E-244	1	NA	
**15**	3.28E-121	1.81E-101	1.54E-54	2.44E-09	3.05E-05	1.38E-57	1.80E-64	4.10E-26	1	1	1	1	1	1	NA
