# Supplementary material for: Computational modeling suggests binding-induced expansion of Epsin disordered regions upon association with AP2
Source: PLoS Comput Biol. 2021 Jan 6;17(1):e1008474. doi: 10.1371/journal.pcbi.1008474 (PMC7787433; doi:10.1371/journal.pcbi.1008474)
Supplement: S9 Text — (PDF) [file pcbi.1008474.s009.pdf]

S9. Mutual information between the occupancy at pairs of binding sites

The hypergeometric tests in Figures 4B-C (main text) were performed to study the independence between the occupancy of pairs of binding motifs. To augment this analysis, we used a second statistical metric – Mutual information (MI). When looking at pairs of events, MI can be used to estimate how much information is conveyed about the second event given information about the first event. Specifically, when looking at the probabilities of AP2α binding at two sites *i* and *j*, the MI is calculated from the probabilities of each site being unbound (denoted by 0) or bound (denoted by 1) using the following table and equation.

State	Site *j* unbound	Site *j* bound
Site *i* unbound	p(*i*=0, *j*=0)	p(*i*=0, *j*=1)
Site *i* bound	p(*i*=1, j=0)	p(*i*=1, *j*=1)

$$MI(i,j) = \sum_{x=0,1} \sum_{y=0,1} p(i = x, j = y) \cdot log \frac{p(i = x, j = y)}{p(i = x)p(j = y)} \qquad (S9.E1)$$

Since the MI value computed using S9.E1 is unbounded, it can be normalized to the range (0,1) to give normalized MI (Norm MI) using the following equations.

$$Norm\ MI(i,j) = \frac{MI(i,j)}{H(i)} \qquad (S9.E2)$$

$$H(i) = -\sum_{x=0,1} p(i = x) \cdot \log\big(p(i = x)\big) \qquad (S9.E3)$$

Where *H(i)* is the entropy of the occupancy of site *i*. When the binding state of one site affects that of another site, a higher Norm MI might be expected. Figure S9.1 shows the Norm MIs values computed for pairs of sites in both Epsin-iDR and Eps15-iDR. (The computation is also repeated for different values of the atom clash thresholds).

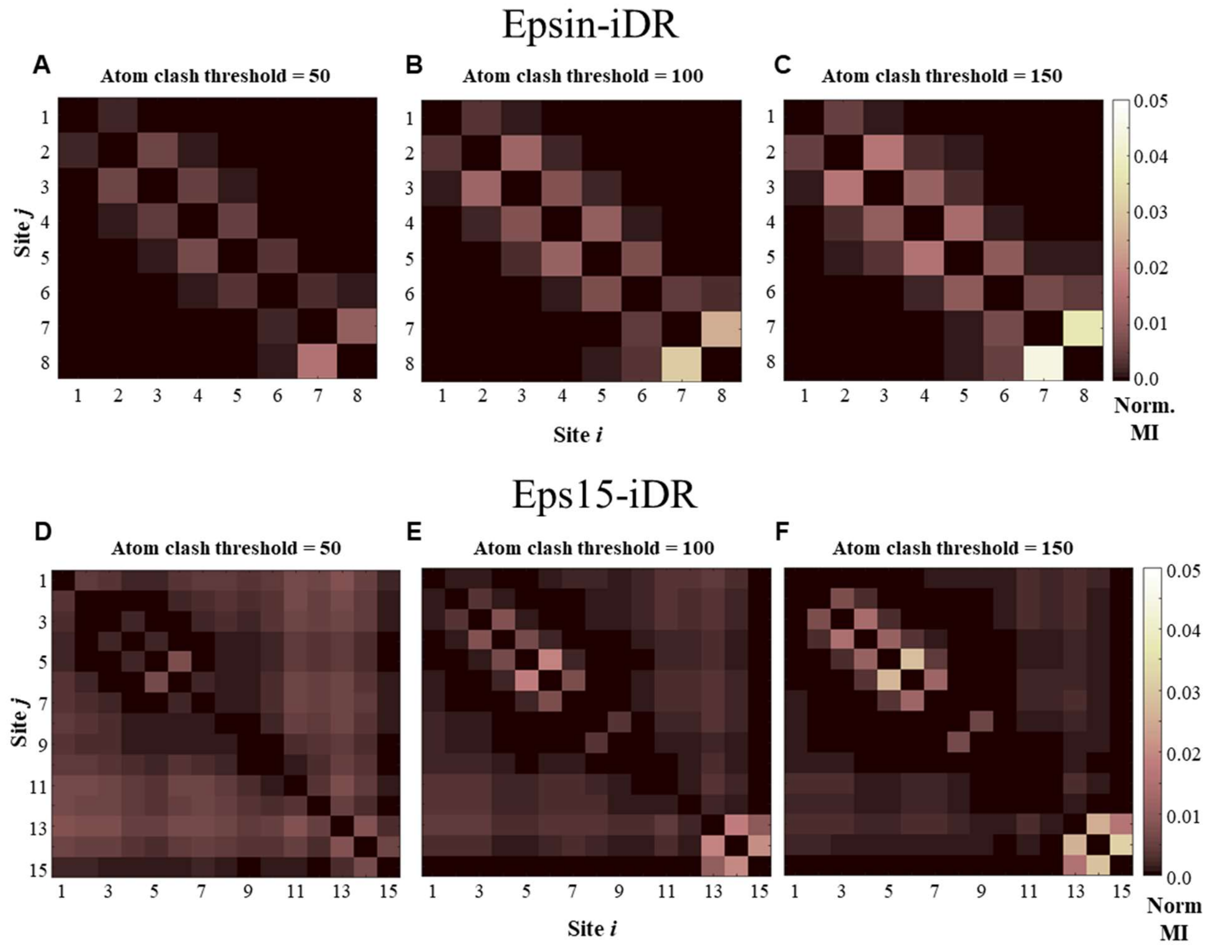

Figure S9.1 Normalized mutual information between the occupancy states of pairs of sites in Epsin-iDR and Eps15-iDR. (A-C) Heatmaps show the Normalized mutual information (Norm MI) between the probability of occupancy of pairs of binding sites in Epsin (using 50, 100 and 150 as clash threshold to permit binding). The Norm MI is high for adjacent pairs of sites and low in the other regions. (D-F) The same computations for Eps15. Unlike Epsin, the Norm MI is high for many pairs of binding sites that are not adjacent in sequence, suggesting that in Eps15, the occupancy state of a binding site can affect the probability of occupancy at sequentially distant binding sites.

For pairs of sites that have non-independent probabilities of occupancy, we next asked whether the correlation is positive or negative – whether occupancy at one site makes the other site more likely or less likely to be occupied. If two binding sites clash due to excluded volume, we would expect to see negative values. If one binding site is cooperative with another, we would expect to see positive values.

While MI does not directly answer this question, MI is a sum of four terms (Equation S9.E1) that have different signs. For instance, if binding at site i increases the probability of binding at site j, the term corresponding to $i = 1, j = 1$ is positive, whereas the term corresponding to $i = 1, j = 0$ is negative. Hence to estimate the direction in which the occupancy of one site affects another site, we computed a metric called Partial mutual information metric (Part MI) as follows.

$$Part\ MI(i,j) = \sum_{x=0,1} p(i = x, j = x) \cdot log \frac{p(i = x, j = x}{p(i = x)p(j = x)} \quad (S9.E4)$$

Part MI is the sum of just two terms as opposed to all four terms. When Part MI is positive, it indicates a positive effect of the occupancy of one site on another, whereas when this term is negative, it indicates that binding at one site adversely affects binding at the other site. Figure 4D-E in the main text shows the computed Part MI values for both Epsin-iDR and Eps15-iDR (computed using an atom clash threshold = 100).
