# Supplementary material for: Computational modeling suggests binding-induced expansion of Epsin disordered regions upon association with AP2
Source: PLoS Comput Biol. 2021 Jan 6;17(1):e1008474. doi: 10.1371/journal.pcbi.1008474 (PMC7787433; doi:10.1371/journal.pcbi.1008474)
Supplement: S10 Text — (PDF) [file pcbi.1008474.s010.pdf]

S10. Sliding window R_G

The frequency of compaction or extension in an IDR would be expected to correlate with the frequency of AP2α binding, because a large protein like AP2α would be accommodated more easily in regions that have extended structure and large R_G. However, the statistics of regional elongation could be more complicated, for example if compaction and extension are affected by factors such as amino acid composition and excluded volume at other locations. Thus, we sought to quantify the relationship between regional elongation and binding site occupancy.

Figures 4C (main text) and Supplement S9 show that in Eps15-iDR, sequentially distant pairs of sites influence the occupancy of each other. To test for corresponding structural differences between bound and unbound ensembles, we performed the following analysis on a random sample of 400,000 structures drawn from each of Epsin-iDR and Eps15-iDR.

For each of the 400,000 structures, the Radius of Gyration (R_G) was computed over a sliding window of 50 amino acids. For the sub-ensemble bound at site 1, for the sub-ensemble bound at site 2, and for each singly-bound sub-ensemble, we categorized the 400,000 structures as members (capable of binding at that site) or non-members (incapable of binding at that site). For each amino acid in the iDR sequence, we compared the distribution of R_G's for the member versus non-member categories, using a Mann-Whitney U test. This analysis gives us a p-value per amino acid, which is then corrected for multiple hypothesis testing using the Benjamini-Hochberg method. Figure S10 shows the corrected p-values for each amino acid along each iDR sequence, comparing members versus non-members of each 1-bound sub-ensemble. Amino acids with $p < 0.05$ indicate that the 50 amino acid R_G (centered around that amino acid) is significantly different in the bound set of conformers, than in the non-members, suggesting local structural changes as a result of AP2α binding.

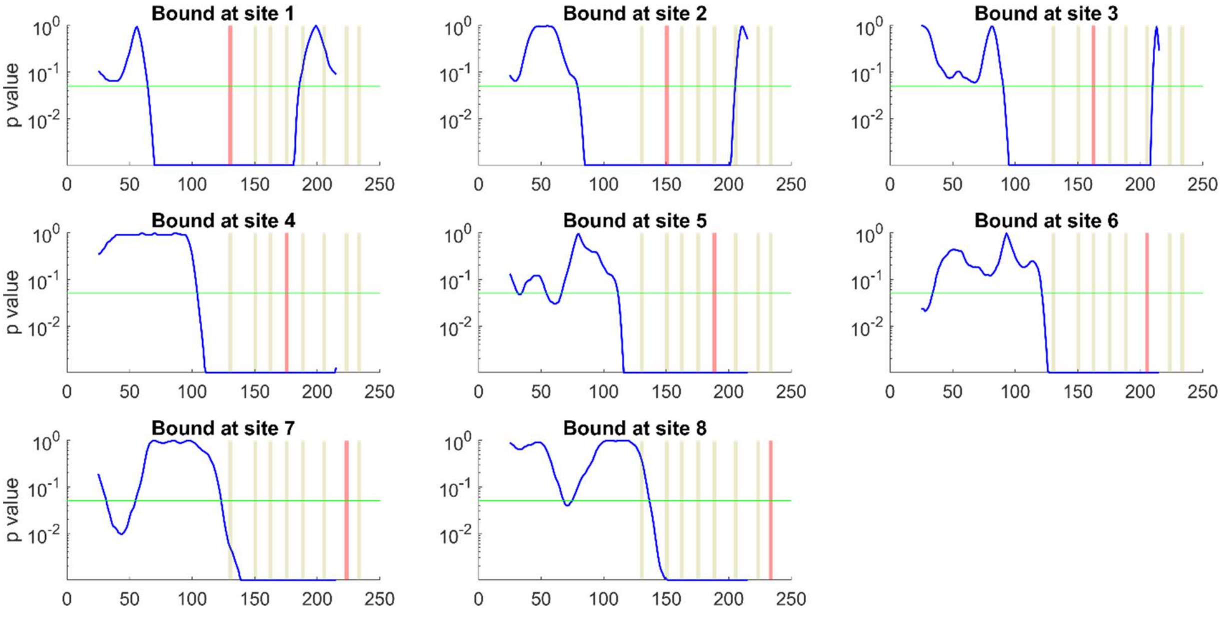

Figure S10.1 Regional Elongation in the Epsin-iDR 1-bound ensemble. p-values comparing the R_G distribution of a 50-aa window of member versus non-member conformers, for each 1-bound sub-ensemble of Epsin-iDR. Each panel represents a specific 1-bound sub-ensemble, e.g., bound at site 1, bound at site 2, etc. The x-axis indicates the amino acid around which a 50-aa window is chosen (25aa to the N-terminus and 24aa to the C-terminus). The y-axis plots the log-scaled, BH-corrected p-values comparing the R_G of the bound versus unbound (member versus non-member) conformers. The vertical yellow bars indicate the positions of the binding motifs. The vertical red bar indicates the site that is bound in the queried sub-ensemble members. The threshold of $p < 0.05$ is marked by a horizontal green line. Note that in the Epsin-iDR, there are statistically significant differences not only around the binding site being queried, but also sometimes at sequentially distant regions. However, these distal regions are not close to other binding sites.

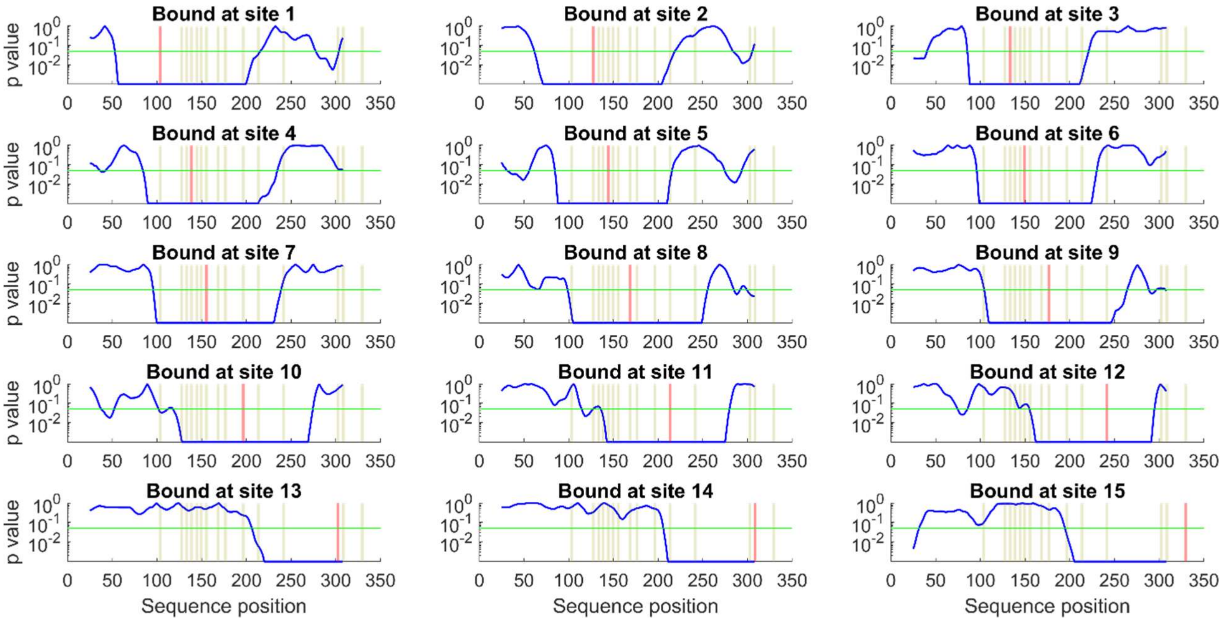

Figure S10.2 Regional Elongation in the Eps15 1-bound ensemble. p-values comparing the R_G distribution of a 50-aa window of member versus non-member conformers, for each 1-bound sub-ensemble of Eps15-iDR. Each panel represents a specific 1-bound sub-ensemble, e.g., bound at site 1, bound at site 2, etc. The x-axis indicates the amino acid around which a 50-aa window is chosen (25aa to the N-terminus and 24aa to the C-terminus). The y-axis plots the log-scaled, BH-corrected p-values comparing the R_G of the bound versus unbound (member versus non-member) conformers. The vertical yellow bars indicate the positions of the binding motifs. The vertical red bar indicates the site that is bound in the queried sub-ensemble members. The threshold of $p < 0.05$ is marked by a horizontal green line. Note that in the Eps15-iDR, there are statistically significant differences not only around the binding site being queried, but also at sequentially distant regions. Unlike with the Eps15-iDR, these distant regions are near other binding sites, suggesting that AP2α binding at one site can cause local conformational changes near other binding sites in the same conformer.
