# Supplementary material for: Computational modeling suggests binding-induced expansion of Epsin disordered regions upon association with AP2
Source: PLoS Comput Biol. 2021 Jan 6;17(1):e1008474. doi: 10.1371/journal.pcbi.1008474 (PMC7787433; doi:10.1371/journal.pcbi.1008474)
Supplement: S11 Text — (PDF) [file pcbi.1008474.s011.pdf]

S11. Distances between sequentially adjacent AP2-binding motifs in Epsin and Eps15 across species.

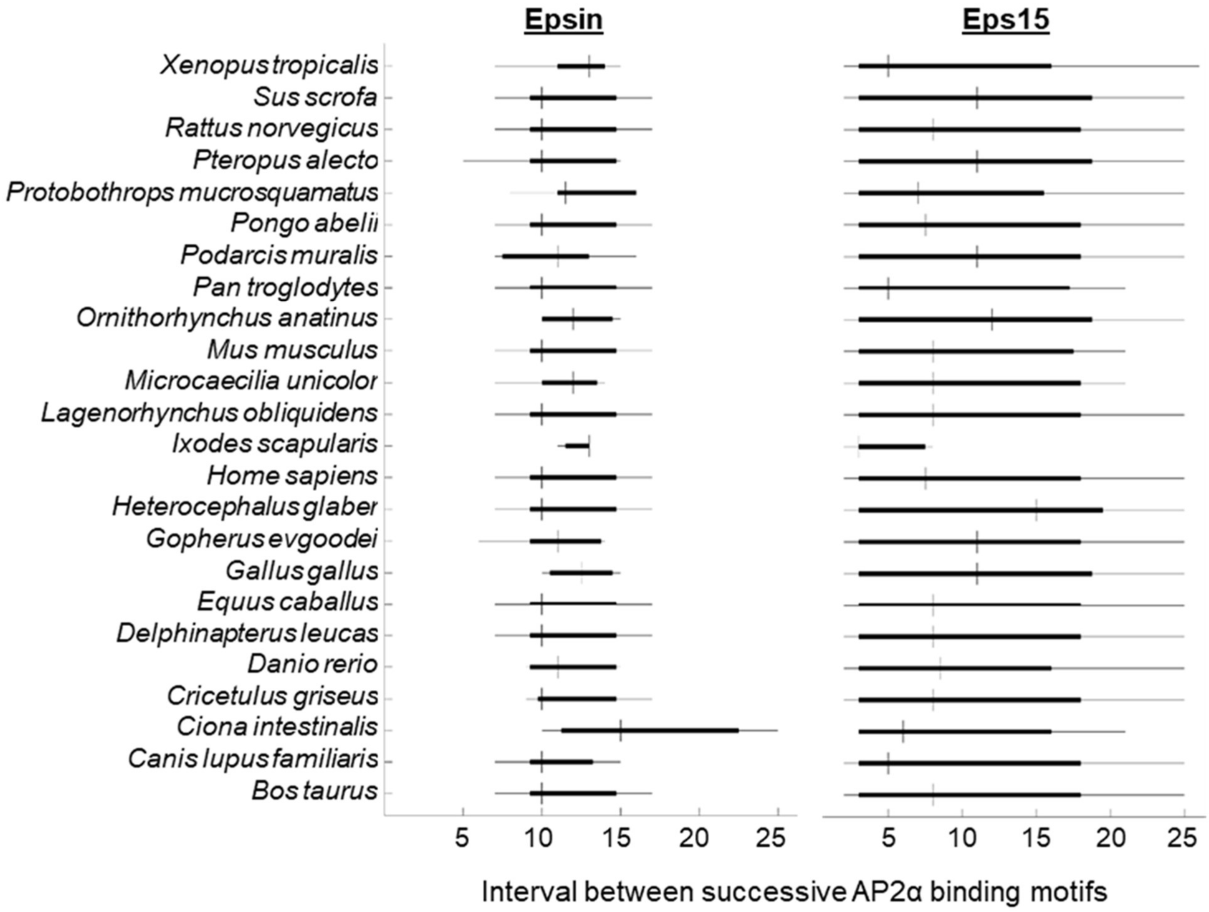

Figure S11.1 Distribution of the intervals between adjacent AP2α binding motifs in Epsin (*left*) and Eps15 (*right*) in multiple species. Thick black horizontal lines represent the inter-quartile range and the vertical black lines represent the median distance between adjacent binding motifs.
